# Treatment-seeking practices of caregivers for children aged less than five years old with diarrhoea in low- and middle-income countries: a systematic review and meta-analysis

**DOI:** 10.7189/jogh.15.04080

**Published:** 2025-04-25

**Authors:** Gedefaw Abeje Fekadu, Muluemebet Abera Wordofa, Firmaye Bogale Woldie, Robera Olana Fite, Kassahun Alemu, Alemayehu Worku, Lisanu Taddesse, Delayehu Bekele, Getachew Tolera, Grace J Chan, Damen Hailemariam

**Affiliations:** 1 Department of Reproductive Health and Population Studies, School of Public Health, College of Medicine and Health Sciences, Bahir Dar University, Bahir Dar, Ethiopia; 2 Health System and Reproductive Health Research Directorate, Ethiopian Public Health Institute, Addis Ababa, Ethiopia; 3 Department of Population and Family Health, Faculty of Public Health, Institute of Health Science, Jimma University, Jimma, Ethiopia; 4 Knowledge Translation Directorate, Ethiopian Public Health Institute, Addis Ababa, Ethiopia; 5 HaSET Maternal and Child Health Research Program, Addis Ababa, Ethiopia; 6 School of Public Health, College of Health Sciences, Addis Ababa University, Addis Ababa, Ethiopia; 7 Department of Obstetrics and Gynaecology, Saint Paul’s Hospital Millennium Medical College, Addis Ababa, Ethiopia; 8 Research and Technology Transfer Directorate, Ethiopian Public Health Institute, Addis Ababa, Ethiopia; 9 Department of Epidemiology, Harvard University T.H. Chan School of Public Health, Boston, Massachusetts, USA; 10 Department of Paediatrics, Boston Children's Hospital, Harvard Medical School, Boston, Massachusetts, USA

## Abstract

**Background:**

Diarrhoeal diseases in children aged <5 years require immediate medical attention. However, previous studies conducted on treatment-seeking practices of caregivers for children aged <5 years with diarrhoea in low- and middle-income countries (LMICs) were inconsistent and inconclusive. We aimed to estimate the pooled treatment-seeking practice of caregivers for children aged <5 years with diarrhoea in LMICs.

**Methods:**

We used the 2020 PRISMA guidelines to conduct this systematic review and meta-analysis. We included both published and unpublished articles in English that reported treatment-seeking practices from health facilities in LMICs between 2010–22. We searched CINAHL, Medline/PubMed, Web of Science, Embase, and grey literature sources. We assessed the eligible articles using the Newcastle-Ottawa Scale quality appraisal checklist and Begg’s test for the presence of publication bias. Further, we used the regression-based Egger test to test for a small study effect. Moreover, we used a narrative synthesis to characterise the studies. We estimated the pooled treatment-seeking practice using a random-effect model. We conducted a subgroup analysis considering the articles' publication status, residence, World Bank income category, study design and approach, and study setting. We presented the results using tables, figures, forest plots, and funnel plots.

**Results:**

We included 76 articles in the analysis. The overall treatment-seeking practices of caregivers were 52.84% (95% confidence interval (CI) = 47.51–58.17). Healthcare-seeking practices in low-income countries (58.12%), lower-middle-income countries (48.41%), and upper-middle-income countries (51.44%) were not statistically different. The pooled treatment-seeking practice for diarrhoea varied by study site: 29.80% (95% CI = 25.00–34.60) in peri-urban, 54.20% (95% CI = 44.71–63.70) in rural, and 47.76% (95% CI = 34.47–61.06) in urban settings. A cross-sectional design was employed in 72 studies, and 71 were quantitative.

**Conclusions:**

Treatment-seeking practice for diarrhoea among children aged <5 years in LMICs remained low. There was no statistically significant difference in treatment-seeking practice for children with diarrhoea by the country's income classification. We recommend further studies to identify factors affecting treatment-seeking practices for diarrhoea among children aged <5 years in LMICs and to act on findings and recommendations.

**Registration:**

PROSPERO: CRD42022290180.

Although preventable and treatable, diarrhoea is the leading cause of morbidity and mortality among children aged <5 years globally [1]. In 2019, diarrhoea was responsible for the deaths of 370 000 children aged <5 years worldwide [2–5]. Diarrhoea is widespread in low- and middle-income countries (LMICs). Children aged <5 years in low-income countries experience an average of three episodes of diarrhoea each year [6].

Diarrheal diseases in children aged <5 years require immediate medical attention. However, in LMICs, the diagnosis and treatment for children aged <5 years with diarrhoea are limited [5]. In addition, the use of health care services by caregivers of children aged <5 years with diarrhoea is determined by caregivers' age, residence, marital status, exposure to media, household wealth index, perceived severity of illness, knowledge of caregivers, educational status, distance from health facilities, geographical barriers to health care access, overall community level literacy, and place of delivery [7–10]. Medical treatment costs are also a burden in some LMICs. When receiving treatment for paediatric diarrhoea, 79% of all costs incurred are direct expenses [11–15].

Previous studies have been conducted in LMICs reporting treatment-seeking behaviour for children aged <5 years with diarrhoea [16–18]. However, the findings reported were inconsistent and inconclusive and did not provide practical recommendations. For example, the treatment-seeking practice ranged from 11.5% in a study conducted in Tanzania [19] to 87% in a study conducted in Niger [20]. The World Health Organization (WHO) recommends gathering evidence on caregivers’ treatment-seeking behaviour, which is critical in informing policy and programs aimed at lowering mortality due to diarrhoea among children aged <5 years [15]. The evidence from this systematic review and meta-analysis can be used by stakeholders, including policymakers and program planners, to improve treatment-seeking practices. Therefore, we aimed to estimate the pooled treatment-seeking practice for diarrhoea among children aged <5 years in LMICs.

## METHODS

### Study design

We used the 2020 PRISMA guidelines [12] while writing this systematic review and meta-analysis. We searched studies using a combination of condition, context, and population criteria. We included research that reported treatment-seeking practices of caregivers for children aged <5 years with diarrhoea from health facilities (public or private hospitals, health centres, health posts, clinics, sentinel health centres) in LMICs. We included articles that originated from LMICs based on the World Bank country and lending groups classification based on gross national income for the fiscal year 2022. We included both quantitative and mixed-method observational studies (cross-sectional, case-control, and cohort studies) based on caregivers’ reports or from health facility records. We searched both published and unpublished articles or reports in English between 2010–22. We excluded articles published before 2010 because of the availability of published systematic reviews between 1990–2010.

### Search strategy and searching sources

We searched multiple databases, including CINAHL, Medline/PubMed, Web of Science, and Embase to access published articles. We searched unpublished articles (grey literature) from Google Scholar, WorldCat, OpenGrey, and online thesis/dissertation repositories. We also screened references of identified articles to identify potential articles that were not found by searching databases. Three independent reviewers (GA, FB, and RO) searched the articles in the databases. We used MeSH keywords and free text search terms to identify the articles. We used the following search terms: diarrh (o)ae, treatment, treatment-seeking, health care seeking, health-seeking behaviour/practice(s), child/children, childhood, under-five, mother, caregiver, developing countries, low-income, middle-income, and the name of each country and combined them with Boolean operators ‘OR’ and ‘AND’ to either broaden or narrow the search (Table S1 in the **Online Supplementary Document**).

### Study selection

We exported all articles to EndNote, version 20.0 (Clarivate Analytics, London, UK) to remove the duplicates and archive. Three authors (GAF, FB, and ROF) reviewed these based on the inclusion and exclusion criteria. Any disagreements between authors were resolved by discussion or by a fourth reviewer (KA).

### Data extraction and processing

We used a standardised pretested data extraction tool that gathered information on author, publication status, study setting, country, LMIC status, study period, urban/rural, study design, sample size, treatment-seeking practice for diarrhoea, sampling methods and methodology. Three independent reviewers (GAF, FB, and ROF) extracted the data. The extracted data was exported to Excel, version 2013 (Microsoft Corporation, Redmond, Washington, USA). We thoroughly checked the data for completeness, and we linked the references to the articles using the unique identifiers provided. We checked the missing data from the articles using the identifier. The clean version of this Excel file was imported into Stata, version 17.0 (StataCorp, College Station, Texas, USA).

### Quality appraisal

We assessed the quality of the included articles using the Newcastle-Ottawa Scale quality appraisal checklist [12]. The scale has a three-domain system, including study group selection, group comparability, and exposure/outcome determination. These domains are divided into eight items, with a maximum possible score of nine. Articles scoring six to nine were rated as good quality, articles scoring three to seven were rated as fair, and those scoring less than three were rated as poor [12].

### Synthesis and analysis

We performed a narrative synthesis to characterise the study participants, study types, study settings, and countries' income classification (Table S2 in the **Online Supplementary Document**).

We used Stata, version 17.0 (StataCorp, College Station, Texas, USA) to analyse the pooled estimate of treatment-seeking practice for diarrhoea in children aged <5 years using a forest plot. To assess homogeneity of study-specific effect sizes, we used the Cochran Q statistic. We used the Q test statistics-related *P*-value to identify any significant heterogeneity between the studies [13]. We used *I^2^* statistics to quantify how much the heterogeneity between studies contributed to the variability in effect-size estimates. Further, we used a random-effects model to assess the pooled treatment-seeking practice for diarrhoea in children aged <5 years [14]. Moreover, we performed a subgroup analysis considering the articles' publication status, place of residence (urban, rural, peri-urban), World Bank income category, study design and approach, and study setting (facility or community-based). We used the test of group difference (Q_b) and associated *P*-value to determine if there was a significant difference in the group-specific overall effect sizes among each subgroup. Moreover, we used the funnel plot to assess the small-study effect. To determine the presence of publication bias, we used Begg’s test for small-study effects and the regression-based Egger test for small-study effects [15]. When determining the presence of publication bias and small-study effects, we used a *P*-value of 0.05.

## RESULTS

**Figure 1:**
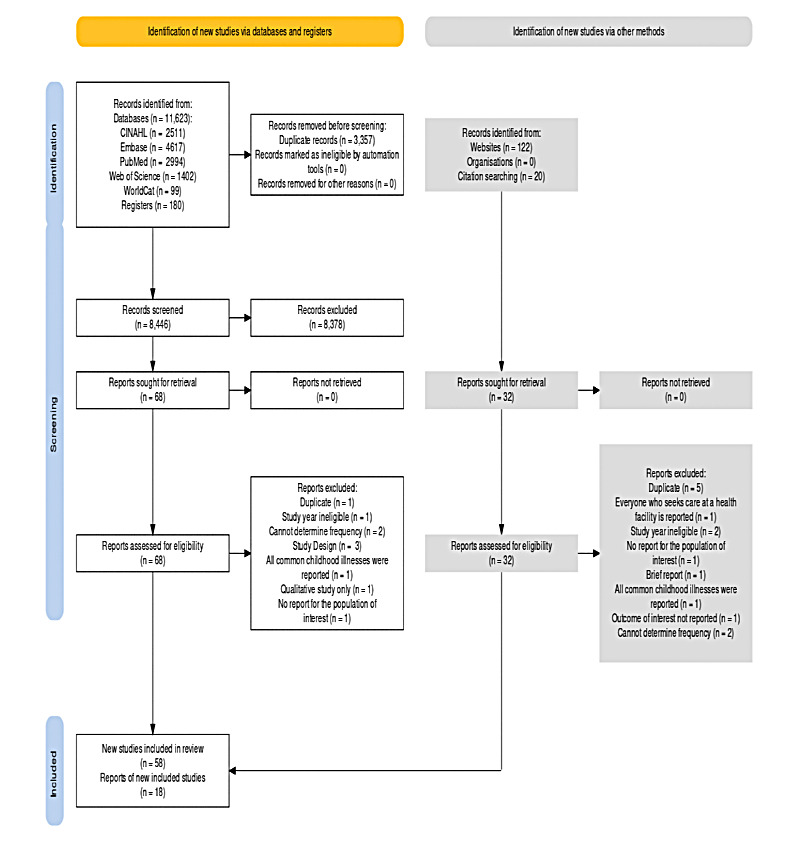
PRISMA 2020 flowchart of article search and selection process to estimate the pooled treatment-seeking practices among caregivers for children aged <5 years with diarrhoea, systematic review and meta-analysis in low- and middle-income countries, 2010–22.

We identified 11 765 articles, including 2511 from CINAHL, 4617 from Embase, 2994 from PubMed, 1402 from Web of Science, and 99 from WorldCat. We discovered 180 articles from other sources, including registers, Google Scholar, and repositories. We removed 3362 because of duplications. After screening the titles and abstracts, we removed totsal 8397 articles. We included the remaining 76 articles in the systematic review and meta-analysis (**Figure 1**).

### Study characteristics

There were 71 published studies and five unpublished articles among the 76 included studies. Three studies [16–18] were conducted in health care facilities, with the remaining 73 conducted in the community. One study was conducted in Albania [21], four in Bangladesh [22–25], one in Botswana [18], one in Burkina Faso [26], one in Burundi [27], two in China [3,4], one in Egypt [28], one in El Salvador [29], 15 in Ethiopia [30–44], two in Gambia [5,6], two in Guatemala [29,45], seven in India [6–12], one in Iran [46], six in Kenya [6,13–17], one in Liberia [47], two in Malawi [18,19], two in Mali [23,48], one in Mexico [29], three in Mozambique [6,20,21], one in Nepal [49], one in Nicaragua [29], one in Niger [50], five in Nigeria [22–26], two in Pakistan [23,51], one in Panama [29], one in Sierra Leone [52], one in Somalia [53], two in Tanzania [54,55], two in Uganda [56,57], two in Zambia [58,59], and one in Zimbabwe [60].

According to the World Bank's income classification, 33 studies were conducted in low-income countries, 38 in lower-middle-income countries [2,6–17,22–37], and five in upper-middle-income countries [3,4,37–39] (Table S2 in the **Online Supplementary Document**). The studies used different study periods ranging from a week to years. However, 10 studies [31,32,35,49,53,56,61–64] did not mention their study period (Table S2 in the **Online Supplementary Document**)

In terms of urban or rural study sites, one study was conducted in peri-urban areas, 21 studies in rural areas [2,5,6,11,15,21,25,34,36-40,45,46,61,65–69], five in semi-urban areas [3,23,24,28,70], 11 in urban areas [24,28,30,42,44,46,48,50,61,71,72], 30 in both urban and rural areas [7-9,13,16-18,20,22,26,27,29,32–35,48–50,63,72–77], and one study in urban, rural, and semi-urban areas [28] (Table S2 in the **Online Supplementary Document**).

In terms of study design, there were three case-control studies [6,12,65], one comparative cross-sectional study [38], and the remaining cross-sectional studies. 71 studies were quantitative, while five were both quantitative and qualitative [2,17,23,45,70].

The mean sample size in these studies was 1500. Study sampling varied with three studies [6,11,14] involving the children. Two studies [19,71] used cluster sampling. One study [16] used consecutive sampling, and one study [27] used convenience sampling. Ten studies [17,25,30,32–35,62,71,76] used multistage sampling. The sampling methods of nine studies [12,19,26,29,35,37,38,61,62] were not specified. One study [73] stated it used proportional allocation. 20 studies used random sampling [2,3,5–7,16,21–23,28,31,32,38,47,61,65,69,70,74,77,78]. Seven studies used stratified cluster sampling [8,9,27,48,49,66,67,72]. Three studies used stratified random sampling [21,30,32] and six studies [13,38,40,45,61,79] used systematic sampling method (Table S2 in the **Online Supplementary Document**).

### Pooled treatment-seeking practice for diarrhoea

**Figure 2:**
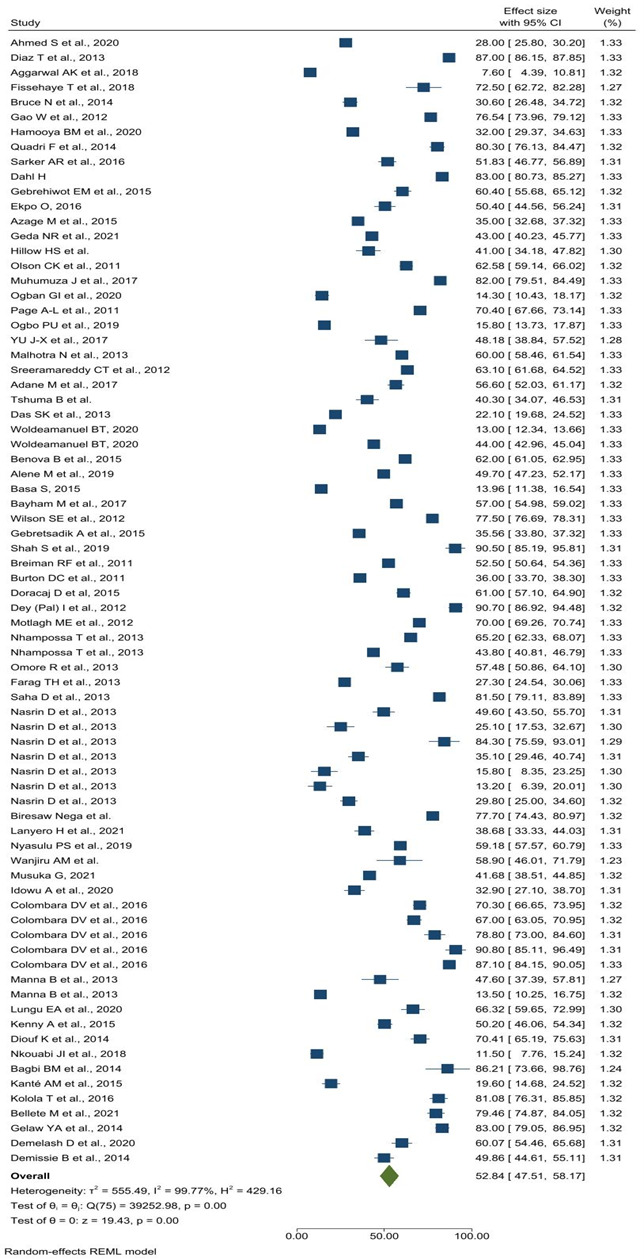
Pooled treatment-seeking practices for caregivers for children aged <5 years with diarrhoea, systematic review and meta-analysis in low- and middle-income countries, 2010–22.

The overall estimated treatment-seeking practices among caregivers for children aged <5 years with diarrhoea in LMICs was 52.84% (95% confidence interval (CI) = 47.51–58.17). *I^2^* of 99.77 indicated that differences between studies account for approximately 99.77% of the variability in effect-size estimates (**Figure 2**).

### Small study effects and publication bias analysis

**Figure 3:**
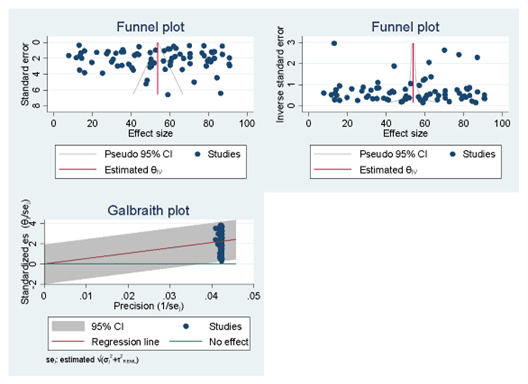
Funnel and Galbraith plots of meta-analysis of the pooled treatment-seeking practices for caregivers for children aged <5 years with diarrhoea, systematic review and meta-analysis in low- and middle-income countries, 2010–22.

Most studies were randomly scattered within the pseudo-95% CI region, and the spread of the observed effect sizes on the estimate of the overall effect size was asymmetric. This indicates the absence of a small-study effect. Similarly, the Galbraith plot indicates that all the effect sizes fall between the no effect and regression lines, indicating that there was no significant heterogeneity because all the studies' effect sizes fall within the 95% CI region (**Figure 3**).

For the null hypothesis test of no small-study effects using the regression-based Egger test and assuming a random-effects model with the residual maximum likelihood estimation method, the magnitude of the small-study effects was 1.17 with a standard error of 2.117 and a Z-score of 0.55 with a *P* < 0.5814. This demonstrates funnel-plot symmetry.

### Subgroup analysis

Treatment-seeking practice was estimated to be 52.45% (95% CI = 46.81–58.10) using published articles and 57.43% (95% CI = 47.81–73.05) using unpublished articles. Using Q statistics revealed that Q = 38 371.88 among published articles and Q = 646.78 among unpublished articles (*P* < 0.001) indicated significant heterogeneity. Similarly, the *I^2^* values for published articles were *I^2^* = 99.79 and for unpublished articles *I^2^* = 98.96, indicating their respective variability in effect-size estimates. The test of group differences (Q_b = 0.34; *P* = 0.56) indicated that the group-specific overall effect sizes were not statistically different.

The test of group (Q_b = 174.50; *P* < 0.001) indicated that the group-specific overall effect sizes among studies were all statistically different: 54.20% in rural areas (95% CI = 44.71–63.7), 47.76% (95% CI = 34.47–61.06) in urban areas, 55.70% (95% CI = 34.47–61.06) in both urban and rural areas, and 62.00% (95% CI = 47.81–63.59) in urban, rural and semi urban areas.

Treatment-seeking practice was estimated to be 58.12% (95% CI = 51.58–64.67) in studies conducted in low-income countries, 48.41% (95% CI = 39.75–57.06) in lower-middle-income countries, and 51.44% (95% CI = 35.47–67.41) in upper-middle-income countries. The presence of between-study heterogeneity was determined using Q statistics, which revealed that Q = 20 892.43 among articles published from low-income countries, Q = 14 887.25 among articles reported from middle-income countries, and Q = 398.61 among articles reported from upper-middle-income countries, with a *P* < 0.001 indicate a significant heterogeneity. Similarly, the *I^2^* values were 99.67 for published articles, 99.81 for articles from lower-middle-income countries, and 98.51 for articles from upper-middle-income countries, indicating that 99.67%, 99.81%, and 98.51% of the variability in effect-size estimates was due to differences in studies between articles in each group, respectively. However, the test of group (Q_b = 3.21; *P* = 0.2) indicated that the group-specific overall effect sizes were not statistically different.

The test of group (Q_b = 96.09; *P* < 0.001) indicated that the group-specific overall effect sizes among studies that were case-control (33.44%; 95% CI = 19.89–46.99), comparative cross-sectional (83.00%; 95% CI = 79.05–86.95), and cross-sectional studies (55.32%; 95% CI = 49.81–60.82), were statistically different (*P* < 0.001). Using the study design of the articles included in this systematic review and meta-analysis, treatment-seeking practices were estimated to be 52.56% (95% CI = 47.12–58.01) using articles that were both qualitative and quantitative and 56.69% (95% CI = 30.11–83.26) using only quantitative studies. The presence of between-study heterogeneity was determined using Q statistics, which revealed that Q = 1453.13 among articles which were both qualitative and quantitative and Q = 31 971.5 among only quantitative studies, with a *P* < 0.001 in both groups indicate significant heterogeneity. Similarly, the *I^2^* values were 99.75 for both groups, indicating that 99.75% of the variability in effect-size estimates is due to differences in studies between articles in each group. However, the test of group differences (Q_b = 0.09; *P* = 0.766) indicated that the group-specific overall effect sizes were not statistically different.

Using the study setting of the systematic review and meta-analysis articles, treatment-seeking practices were estimated to be 39.74% (95% CI = 12.83–66.65) from articles conducted at health facilities and 53.58% (95% CI = 47.94–58.82) from articles conducted at the community level. The Q = 1373.05 among articles conducted at health facilities and the Q = 37 763.86 among articles conducted at the community level, with a *P* < 0.001 in both groups, indicate significant heterogeneity. Similarly, the *I^2^* values were 99.74 for published articles and 99.76 for unpublished articles, indicating that 99.74% and 98.76% of the variability in effect-size estimates was due to differences in studies between articles in each group, respectively. However, the test of group differences (Q_b = 0.95; *P* = 0.330) indicate that the group-specific overall effect sizes were not statistically different.

## DISCUSSION

The estimated pooled proportion of treatment-seeking practices of caregivers for children aged <5 years with diarrhoea in LMICs was 52.84%. Disaggregated by income levels, the difference in caregiver health care-seeking practices in low-income countries (58.12%), in lower-middle-income countries (48.41%), and in upper-middle-income countries (51.44%) was not statistically different.

In LMICs, caregivers of just over half (52.84%) of children aged <5 years with diarrhoea sought treatment at health facilities. This figure is expected in LMICs due to the inaccessibility of health facilities [44,80,81], lower level of integrated primary care services [82], limitations in the implementation of child health education and social services [83], use of traditional medicines and practices [84,85], and use of dietary management of diarrhoea [86]. The overall effect size was statistically significant in the analysis, which implies the presence of significant heterogeneity between the studies. Additionally, the Q test also indicated the presence of significant heterogeneity between the studies included in this systematic review and meta-analysis. The heterogeneity may be due to the difference in the time the surveys were conducted and the difference in the study design.

The group-specific overall effect sizes among studies that were conducted in rural, urban, both urban and rural, and urban, rural, and semi-urban areas were statistically different. A study identified that the site of the study was shown to have an impact on the treatment-seeking practices for childhood diarrhoea [87]. The caregivers' economic status has also been reported to be related to the residential area, which influences the use of health care services [88]. Furthermore, geographic factors have been known to influence access to health care and the number of health providers available to care for children in various communities [89].

Similarly, the group-specific overall effect sizes among different study designs (case-control, comparative cross-sectional, and cross-sectional studies) were statistically different. Methodological heterogeneity is frequently assumed to be one of the factors contributing to heterogeneity among studies included in a meta-analysis [90,91]. We conducted a subgroup analysis to show the variation by study design.

In this systematic review and meta-analysis, we maintained a high quality of study selection and analysis. We evaluated the selected articles using a valid quality assessment tool before including them in the review. The funnel plot revealed a symmetrical distribution, revealing the small-study effect. Furthermore, small-study effects were assessed using the regression-based Egger test. One limitation was that although we included articles from multiple countries, the number of studies from some geographical areas was limited. Therefore, the results of this subgroup analysis should be interpreted with caution. In addition, this review did not address factors associated with treatment-seeking practice.

## CONCLUSIONS

Treatment-seeking practice for diarrhoea among children aged <5 years in LMICs remained low. Overall, more efforts are needed to improve treatment-seeking practices. We recommend further studies to identify factors affecting treatment-seeking practices for diarrhoea among children aged <5 years in LMICs.

## Additional material


Online Supplementary Document

## References

[1] Hartman RM Cohen AL Antoni S Mwenda J Weldegebriel G Biey J Risk Factors for Mortality Among Children Younger Than Age 5 Years With Severe Diarrhea in Low- and Middle-income Countries: Findings From the World Health Organization-coordinated Global Rotavirus and Pediatric Diarrhea Surveillance Networks. Clin Infect Dis. 2023;76:e1047–53. 10.1093/cid/ciac561 35797157 PMC9907489

[2] World Health Organization. Diarrhoeal disease. Key facts. 2024. Available: https://www.who.int/news-room/fact-sheets/detail/diarrhoeal-disease. Accessed: 17 April 2025.

[3] Fagbamigbe AF Uthman AO Ibisomi L Hierarchical disentanglement of contextual from compositional risk factors of diarrhoea among under-five children in low- and middle-income countries. Sci Rep. 2021;11:8564. 10.1038/s41598-021-87889-2 33879839 PMC8058334

[4] Wolf J Hubbard S Brauer M Ambelu A Arnold BF Bain R Effectiveness of interventions to improve drinking water, sanitation, and handwashing with soap on risk of diarrhoeal disease in children in low-income and middle-income settings: a systematic review and meta-analysis. Lancet. 2022;400:48–59. 10.1016/S0140-6736(22)00937-0 35780792 PMC9251635

[5] Bradley SEK Rosapep L Shiras T Where Do Caregivers Take Their Sick Children for Care? An Analysis of Care Seeking and Equity in 24 USAID Priority Countries. Glob Health Sci Pract. 2020;8:518–33. 10.9745/GHSP-D-20-00115 33008861 PMC7541105

[6] World Health Organization. Health topics: Diarrhoea. 2024. Available: https://www.who.int/health-topics/diarrhoea#tab=tab_1. Accessed: 17 April 2025.

[7] Bogler L Weber AC Ntambi J Simen-Kapeu A Zagre NM Ekpini RE Health-care seeking for childhood diseases by parental age in Western and Central Africa between 1995 and 2017: A descriptive analysis using DHS and MICS from 23 low- and middle-income countries. J Glob Health. 2021;11:13010. 10.7189/jogh.11.13010 34484717 PMC8397328

[8] Harb A Abraham S O’Dea M Hantosh HA Jordan D Habib I Sociodemographic Determinants of Healthcare-Seeking Options and Alternative Management Practices of Childhood Diarrheal Illness: A Household Survey among Mothers in Iraq. Am J Trop Med Hyg. 2020;104:748–55. 10.4269/ajtmh.20-0529 33289474 PMC7866356

[9] Adeoti IG Cavallaro FL Determinants of care-seeking behaviour for fever, acute respiratory infection and diarrhoea among children under five in Nigeria. PLoS One. 2022;17:e0273901. 10.1371/journal.pone.0273901 36107948 PMC9477346

[10] Zenebe GA Gebretsadik S Muche T Sisay D Meno A Hareru HE Level of Mothers’/Caregivers’ Healthcare-Seeking Behavior for Child’s Diarrhea, Fever, and Respiratory Tract Infections and Associated Factors in Ethiopia: A Systematic Review and Meta-Analysis. BioMed Res Int. 2022;2022:4053085. 10.1155/2022/4053085 35898685 PMC9314182

[11] Baral R Nonvignon J Debellut F Agyemang SA Clark A Pecenka C Cost of illness for childhood diarrhea in low- and middle-income countries: a systematic review of evidence and modelled estimates. BMC Public Health. 2020;20:619. 10.1186/s12889-020-08595-8 32370763 PMC7201538

[12] Peterson J, Welch V, Losos M, Tugwell P. The Newcastle-Ottawa scale (NOS) for assessing the quality of nonrandomised studies in meta-analyses. 2011. Available: https://www.ohri.ca/programs/clinical_epidemiology/oxford.asp. Accessed: 17 April 2025.

[13] Sedgwick P Meta-analyses: what is heterogeneity? BMJ. 2015;350:h1435. 10.1136/bmj.h1435 25778910

[14] Dettori JR Norvell DC Chapman JR Fixed-Effect vs Random-Effects Models for Meta-Analysis: 3 Points to Consider. Global Spine J. 2022;12:1624–6. 10.1177/21925682221110527 35723546 PMC9393987

[15] Lin L Chu H Murad MH Hong C Qu Z Cole SR Empirical Comparison of Publication Bias Tests in Meta-Analysis. J Gen Intern Med. 2018;33:1260–7. 10.1007/s11606-018-4425-7 29663281 PMC6082203

[16] Ogbo P Ainaf B Owoborode O The Prescription of Medicines for Childhood Acute Diarrhoea: A Retrospective Study at Four Secondary Healthcare Facilities in Lagos State, Nigeria. Nigerian Journal of Pharmaceutical Research. 2022;17:257–65. 10.4314/njpr.v17i2.11

[17] Sreeramareddy CT Sathyanarayana TN Harsha Kumar HN Utilization of Health Care Services for Childhood Morbidity and Associated Factors in India: A National Cross-Sectional Household Survey. PLoS One. 2012;7:e51904. 10.1371/journal.pone.0051904 23284810 PMC3526528

[18] Mukiira C Ibisomi L Health care seeking practices of caregivers of children under 5 with diarrhea in two informal settlements in Nairobi, Kenya. J Child Health Care. 2015;19:254–64. 10.1177/1367493513508231 24270995

[19] Doracaj D Grabocka E Hallkaj E Vyshka G Healthcare-seeking practices for common childhood illnesses in northeastern Albania: A community-based household survey. J Adv Med Pharm Sci. 2015;3:31–41. 10.9734/JAMPS/2015/16088

[20] Sabbir A Yunus FM Healthcare seeking behavior for common illness among Bangladeshi under-five children: a nationwide cross-sectional survey. Child Youth Serv Rev. 2020;119:105644. 10.1016/j.childyouth.2020.105644

[21] Sarker AR Sultana M Mahumud RA Sheikh N Van Der Meer R Morton A Prevalence and health care–seeking behavior for childhood diarrheal disease in Bangladesh. Glob Pediatr Health. 2016;3:X16680901. 10.1177/2333794X16680901 28229092 PMC5308522

[22] Das SK Chisti MJ Huq S Malek MA Vanderlee L Kaur G Clinical characteristics, etiology and antimicrobial susceptibility among overweight and obese individuals with diarrhea: observed at a large diarrheal disease hospital, Bangladesh. PLoS One. 2013;8:e70402. 10.1371/journal.pone.0070402 23936424 PMC3731266

[23] Nasrin D Wu Y Blackwelder WC Farag TH Saha D Sow SO Health care seeking for childhood diarrhea in developing countries: evidence from seven sites in Africa and Asia. Am J Trop Med Hyg. 2013;89:3–12. 10.4269/ajtmh.12-0749 23629939 PMC3748499

[24] Wilson SE Ouédraogo CT Prince L Ouédraogo A Hess SY Rouamba N Caregiver recognition of childhood diarrhea, care seeking behaviors and home treatment practices in rural Burkina Faso: a cross-sectional survey. PLoS One. 2012;7:e33273. 10.1371/journal.pone.0033273 22428006 PMC3302832

[25] Diouf K Tabatabai P Rudolph J Marx M Diarrhoea prevalence in children under five years of age in rural Burundi: an assessment of social and behavioural factors at the household level. Glob Health Action. 2014;7:24895. 10.3402/gha.v7.24895 25150028 PMC4141944

[26] Gao W Dang S Yan H Wang D Care-seeking pattern for diarrhea among children under 36 months old in rural western China. PLoS One. 2012;7:e43103. 10.1371/journal.pone.0043103 22912799 PMC3422327

[27] Yu JX Zhu WP Ye CC Xue CY Lai SJ Zhang HL A cross-sectional study of acute diarrhea in Pudong, Shanghai, China: prevalence, risk factors, and healthcare-seeking practices. Epidemiol Infect. 2017;145:2735–44. 10.1017/S0950268817001844 28830575 PMC9148769

[28] Benova L Campbell OM Ploubidis GB Socio-economic inequalities in curative health-seeking for children in Egypt: analysis of the 2008 Demographic and Health Survey. BMC Health Serv Res. 2015;15:482. 10.1186/s12913-015-1150-3 26496850 PMC4619580

[29] Colombara DV Hernández B McNellan CR Desai SS Gagnier MC Haakenstad A Diarrhea Prevalence, Care, and Risk Factors Among Poor Children Under 5 Years of Age in Mesoamerica. Am J Trop Med Hyg. 2016;94:544–52. 10.4269/ajtmh.15-0750 26787152 PMC4775889

[30] Gebrehiwot EM Berheto TM Worku A Darebo TD Sibamo EL Childhood diarrhea in Central Ethiopia: determining factors for mothers in seeking modern health treatments. Science Journal of Clinical Medicine. 2015;4:4–9. 10.11648/j.sjcm.20150401.12

[31] Azage M Haile D Factors affecting healthcare service utilization of mothers who had children with diarrhea in Ethiopia: evidence from a population based national survey. Rural Remote Health. 2015;15:3493. 10.22605/RRH3493 26732052

[32] Geda NR Feng CX Whiting SJ Lepnurm R Henry CJ Janzen B Disparities in mothers’ healthcare seeking behavior for common childhood morbidities in Ethiopia: based on nationally representative data. BMC Health Serv Res. 2021;21:670. 10.1186/s12913-021-06704-w 34238320 PMC8265080

[33] Alene M Yismaw L Berelie Y Kassie B Health care utilization for common childhood illnesses in rural parts of Ethiopia: evidence from the 2016 Ethiopian demographic and health survey. BMC Public Health. 2019;19:57. 10.1186/s12889-019-6397-x 30642301 PMC6332529

[34] Gebretsadik A Worku A Berhane Y Less Than One-Third of Caretakers Sought Formal Health Care Facilities for Common Childhood Illnesses in Ethiopia: Evidence from the 2011 Ethiopian Demographic Health Survey. Int J Family Med. 2015;2015:516532. 10.1155/2015/516532 26273479 PMC4529949

[35] Nega B, Bogale KA, Nigussie ZM. Health care seeking behavior and associated factor among mothers/caregivers of under-five children with acute diarrhea in Dangila zuria Woreda, North west Ethiopia. BioRxiv: 667923 [preprint]. 2019. Available: https://www.biorxiv.org/content/10.1101/667923v1. Accessed: 15 April 2025. 10.1101/667923

[36] Kolola T Gezahegn T Addisie M Health Care Seeking Behavior for Common Childhood Illnesses in Jeldu District, Oromia Regional State, Ethiopia. PLoS One. 2016;11:e0164534. 10.1371/journal.pone.0164534 27741273 PMC5065207

[37] Demissie B Ejie B Zerihun H Tafese Z Gamo G Tafese T Assessment of health care seeking behavior of caregivers for common childhood illnesses in Shashogo Woreda, Southern Ethiopia. Ethiop J Health Dev. 2014;28:36–43.

[38] Gelaw YA Biks GA Alene KA Effect of residence on mothers’ health care seeking behavior for common childhood illness in Northwest Ethiopia: a community based comparative cross–sectional study. BMC Res Notes. 2014;7:705. 10.1186/1756-0500-7-705 25297952 PMC4210615

[39] Demelash D Mebratu W Health care seeking behavior of mothers for common childhood illness and associated factors in Woldia town administration, Northeast Ethiopia. Fam Med Med Sci Res. 2020;9:256.

[40] Saha D Akinsola A Sharples K Adeyemi MO Antonio M Imran S Health Care Utilization and Attitudes Survey: understanding diarrheal disease in rural Gambia. Am J Trop Med Hyg. 2013;89:13–20. 10.4269/ajtmh.12-0751 23629926 PMC3748496

[41] Adane M Mengistie B Mulat W Kloos H Medhin G Utilization of health facilities and predictors of health-seeking behavior for under-five children with acute diarrhea in slums of Addis Ababa, Ethiopia: a community-based cross-sectional study. J Health Popul Nutr. 2017;36:9. 10.1186/s41043-017-0085-1 28376916 PMC5381138

[42] Bellete M Boke MM Yenit MK Child Caregiver’s healthcare seeking behavior and its determinants for common childhood illnesses in Addis Ababa, Ethiopia: a community-based study. Ital J Pediatr. 2021;47:99. 10.1186/s13052-021-01049-w 33882994 PMC8058976

[43] Woldeamanuel BT Trends and Factors Associated with Healthcare Utilization for Childhood Diarrhea and Fever in Ethiopia: Further Analysis of the Demographic and Health Surveys from 2000 to 2016. J Environ Public Health. 2020;2020:8076259. 10.1155/2020/8076259 32148530 PMC7049399

[44] Peters DH Garg A Bloom G Walker DG Brieger WR Rahman MH Poverty and access to health care in developing countries. Ann N Y Acad Sci. 2008;1136:161–71. 10.1196/annals.1425.011 17954679

[45] Bruce N Pope D Arana B Shiels C Romero C Klein R Determinants of care seeking for children with pneumonia and diarrhea in Guatemala: implications for intervention strategies. Am J Public Health. 2014;104:647–57. 10.2105/AJPH.2013.301658 24524510 PMC4025722

[46] Motlagh ME Heidarzadeh A Hashemian H Dosstdar M Patterns of Care Seeking During Episodes of Childhood Diarrhea and its Relation to Preventive Care Patterns: National Integrated Monitoring and Evaluation Survey (IMES) of Family Health. Islamic Republic of Iran. Int J Prev Med. 2012;3:60–7. 22355479 PMC3278871

[47] Kenny A Basu G Ballard M Griffiths T Kentoffio K Niyonzima JB Remoteness and maternal and child health service utilization in rural Liberia: A population-based survey. J Glob Health. 2015;5:020401. 10.7189/jogh.05.020401 26207180 PMC4512264

[48] Farag TH Kotloff KL Levine MM Onwuchekwa U Van Eijk AM Doh S Seeking care for pediatric diarrheal illness from traditional healers in Bamako, Mali. Am J Trop Med Hyg. 2013;89:21–8. 10.4269/ajtmh.12-0753 23629935 PMC3748497

[49] Shah S Shreshta M Sharma B Pandey N Dahal S Knowledge and Practice on Childhood Diarrhea among Mothers having Children Under Five Years of Age in Madhuban, Sunsari-Nepal. Religion. 2019;20:29.

[50] Page A-L Hustache S Luquero FJ Djibo A Manzo ML Grais RF Health care seeking behavior for diarrhea in children under 5 in rural Niger: results of a cross-sectional survey. BMC Public Health. 2011;11:389–95. 10.1186/1471-2458-11-389 21612640 PMC3121637

[51] Quadri F Nasrin D Khan A Bokhari T Tikmani SS Nisar MI Health care use patterns for diarrhea in children in low-income periurban communities of Karachi, Pakistan. Am J Trop Med Hyg. 2013;89:49–55. 10.4269/ajtmh.12-0757 23629928 PMC3748501

[52] Diaz T George AS Rao SR Bangura PS Baimba JB McMahon SA Healthcare seeking for diarrhoea, malaria and pneumonia among children in four poor rural districts in Sierra Leone in the context of free health care: results of a cross-sectional survey. BMC Public Health. 2013;13:157. 10.1186/1471-2458-13-157 23425576 PMC3598532

[53] Hillow HS. Caregivers Knowledge and Practices in Management of diarrhoea among children aged 6-59 months in Ceelafweyn District, Sanag Region, Somaliland [master thesis]. Nairoby, Kenya: Kenyatta University; 2018. Available: https://ir-library.ku.ac.ke/server/api/core/bitstreams/fe1bc66f-64ea-410f-bcc1-5c1dbb3ea2fa/content. Accessed: 17 April 2025.

[54] Nkouabi JI Mchaile DN Msuya SE Prevalence, Health Care Seeking Behaviour and Factors Associated with Home Management of Diarrhoea Among Caregivers with Children Aged 2-59 Months, Attending Health Facilities in Moshi Municipality: A Hospital Based Cross-Sectional Study. J Trop Med Health. 2017;2017:110.

[55] Kanté AM Gutierrez HR Larsen AM Jackson EF Helleringer S Exavery A Childhood Illness Prevalence and Health Seeking Behavior Patterns in Rural Tanzania. BMC Public Health. 2015;15:951. 10.1186/s12889-015-2264-6 26399915 PMC4581421

[56] Muhumuza J Muhirwe LB Ssentamu C Conteh MM Dunne NM Karumuna R Factors influencing timely response to health care seeking for diarrheal episodes among children under five by caregivers in rural Uganda. Science Journal of Public Health. 2017;5:246–53. 10.11648/j.sjph.20170503.23

[57] Lanyero H Ocan M Obua C Lundborg CS Nanzigu S Katureebe A Antibiotic use among children under five years with diarrhea in rural communities of Gulu, northern Uganda: a cross-sectional study. BMC Public Health. 2021;21:1254. 10.1186/s12889-021-11254-1 34187421 PMC8244156

[58] Hamooya BM Masenga SK Halwiindi H Predictors of diarrhea episodes and treatment-seeking behavior in under-five children: a longitudinal study from rural communities in Zambia. Pan Afr Med J. 2020;36:115. 32821326 10.11604/pamj.2020.36.115.20180 PMC7406458

[59] Dahl H. Mothers’ treatment seeking behavior for children with diarrhea: a cross-sectional study in Zambia. Sundsvall, Sweden: Mid Sweden University; 2021. Available: https://www.diva-portal.org/smash/get/diva2:1539169/FULLTEXT01.pdf. Accessed: 17 April 2025.

[60] Musuka G Dzinamarira T Murewanhema G Cuadros D Chingombe I Herrera H Associations of diarrhea episodes and seeking medical treatment among children under five years: Insights from the Zimbabwe Demographic Health Survey (2015-2016). Food Sci Nutr. 2021;9:6335–42. 10.1002/fsn3.2596 34760263 PMC8565232

[61] Pal ID Chaudhuri R Acute Childhood Illnesses and Health Seeking Behaviour among under five children in a village of Hooghly district, West Bengal. IJMEDPH. 2012;2:5530.

[62] Wanjiru AM. Caregiver’s knowledge, perceptions and practices on diarrheoal diseases among children under five years in Turkana County, Kenya [dissertation]. Nairobi, Kenya: Kenyatta University; 2018. Available: https://ir-library.ku.ac.ke/server/api/core/bitstreams/1a50549e-1285-4f15-a431-05ca3ed5331b/content. Accessed: 17 April 2025.

[63] Ekpo O Careseeking for childhood diarrhoea at the primary level of care in communities in Cross River State, Nigeria. J Epidemiol Glob Health. 2016;6:303–13. 10.1016/j.jegh.2016.08.002 27639039 PMC7320457

[64] Bagbi BM Obieche A Enato E Assessment of care-seeking behaviour for under five years old children with malaria and other childhood illnesses in some communities in Edo State, Nigeria. Journal of Science and Practice of Pharmacy. 2014;1:49–53.

[65] Fissehaye T Damte A Fantahun A Gebrekirstos K Health care seeking behaviour of mothers towards diarrheal disease of children less than 5 years in Mekelle city, North Ethiopia. BMC Res Notes. 2018;11:749. 10.1186/s13104-018-3850-3 30348211 PMC6196468

[66] Aggarwal AK Dalpath S Das D Singh A Anand H Kaur K Treatment Seeking Practices for Diarrhea and Acute Respiratory Infections in Haryana: Need to Curb High Antibiotic Use. International Journal of Health Systems and Implementation Research. 2018;2:28–38.

[67] Malhotra N Upadhyay RP Why are there delays in seeking treatment for childhood diarrhoea in India? Acta Paediatr. 2013;102:e413–8. 10.1111/apa.12304 23718747

[68] Basa S Prevalence of Diarrhoea among Under-Five children and Health Seeking Behaviour of their Mothers in an Urban Slum of Delhi. Asian Journal of Biomedical and Pharmaceutical Sciences. 2015;5:08–11. 10.15272/ajbps.v5i45.701

[69] Manna B Nasrin D Kanungo S Roy S Ramamurthy T Kotloff KL Determinants of health care seeking for diarrheal illness in young children in urban slums of Kolkata, India. Am J Trop Med Hyg. 2013;89:56–61. 10.4269/ajtmh.12-0756 23629936 PMC3748502

[70] Olson CK Blum LS Patel KN Oria PA Feikin DR Laserson KF Community case management of childhood diarrhea in a setting with declining use of oral rehydration therapy: findings from cross-sectional studies among primary household caregivers, Kenya, 2007. Am J Trop Med Hyg. 2011;85:1134–40. 10.4269/ajtmh.2011.11-0178 22144458 PMC3225166

[71] Burton DC Flannery B Onyango B Larson C Alaii J Zhang X Healthcare-seeking behaviour for common infectious disease-related illnesses in rural Kenya: a community-based house-to-house survey. J Health Popul Nutr. 2011;29:61–70. 10.3329/jhpn.v29i1.7567 21528791 PMC3075057

[72] Bayham M Blevins M Lopez M Olupona O González-Calvo L Ndatimana E Predictors of Health-Care Utilization Among Children 6-59 Months of Age in Zambézia Province, Mozambique. Am J Trop Med Hyg. 2017;96:493–500. 10.4269/ajtmh.16-0233 27821686 PMC5303059

[73] Nyasulu PS Ngamasana E Kandala N-B Sources of health care among under-5 Malawian children with diarrhea episodes: an analysis of the 2017 demographic and health survey. Glob Pediatr Health. 2019;6:X19855468. 10.1177/2333794X19855468 31259208 PMC6589950

[74] Lungu EA Darker C Biesma R Determinants of healthcare seeking for childhood illnesses among caregivers of under-five children in urban slums in Malawi: a population-based cross-sectional study. BMC Pediatr. 2020;20:20. 10.1186/s12887-020-1913-9 31952484 PMC6966883

[75] Nhampossa T Mandomando I Acacio S Nhalungo D Sacoor C Nhacolo A Health care utilization and attitudes survey in cases of moderate-to-severe diarrhea among children ages 0–59 months in the District of Manhica, southern Mozambique. Am J Trop Med Hyg. 2013;89:41. 10.4269/ajtmh.12-0754 23629927 PMC3748500

[76] Ogban G Ndueso E Iwuafor A Emanghe U Ushie S Ejemot-Nwadiaro R Basic Knowledge of Childhood Diarrhea and Health-seeking Practices of Caregivers of Under-five Childrenin Calabar-South, Calabar, Nigeria. Asian Journal of Medicine and Health. 2020;18:12–23. 10.9734/ajmah/2020/v18i430195

[77] Idowu A Olasinde Y Aremu AO Israel OK Ala OA Sociodemographic factors associated with utilization of oral rehydration therapy among under five children with diarrhoea in a rural Nigerian community. Anatol J Fam Med. 2020;3:221. 10.5505/anatoljfm.2020.96636

[78] Omore R O’Reilly CE Williamson J Moke F Were V Farag TH Health care-seeking behavior during childhood diarrheal illness: results of health care utilization and attitudes surveys of caretakers in western Kenya, 2007-2010. Am J Trop Med Hyg. 2013;89:29–40. 10.4269/ajtmh.12-0755 23629929 PMC3748498

[79] Breiman RF Olack B Shultz A Roder S Kimani K Feikin DR Healthcare-use for major infectious disease syndromes in an informal settlement in Nairobi, Kenya. J Health Popul Nutr. 2011;29:123–33. 10.3329/jhpn.v29i2.7854 21608421 PMC3126984

[80] Bright T Felix L Kuper H Polack S A systematic review of strategies to increase access to health services among children in low- and middle-income countries. BMC Health Serv Res. 2017;17:252. 10.1186/s12913-017-2180-9 28381276 PMC5382494

[81] Hunter AA Flores G Social determinants of health and child maltreatment: a systematic review. Pediatr Res. 2021;89:269–74. 10.1038/s41390-020-01175-x 32977325

[82] Druetz T Integrated primary health care in low- and middle-income countries: a double challenge. BMC Med Ethics. 2018;19:48. 10.1186/s12910-018-0288-z 29945623 PMC6020002

[83] Kruk ME Lewis TP Arsenault C Bhutta ZA Irimu G Jeong J Improving health and social systems for all children in LMICs: structural innovations to deliver high-quality services. Lancet. 2022;399:1830–44. 10.1016/S0140-6736(21)02532-0 35489361 PMC9077444

[84] Njume C Goduka NI Treatment of diarrhoea in rural African communities: an overview of measures to maximise the medicinal potentials of indigenous plants. Int J Environ Res Public Health. 2012;9:3911–33. 10.3390/ijerph9113911 23202823 PMC3524604

[85] Maroyi A Treatment of diarrhoea using traditional medicines: contemporary research in South Africa and Zimbabwe. Afr J Tradit Complement Altern Med. 2016;13:5–10. 10.21010/ajtcam.v13i6.2 28480353 PMC5412201

[86] Gaffey MF Wazny K Bassani DG Bhutta ZA Dietary management of childhood diarrhea in low- and middle-income countries: a systematic review. BMC Public Health. 2013;13:S17. 10.1186/1471-2458-13-S3-S17 24564685 PMC3847348

[87] Bennett A, Eisele T, Keating J, Yukich J. Global trends in care seeking and access to diagnosis and treatment of childhood illnesses. Rockville, Maryland, USA: ICF International; 2015.

[88] Allensworth DD Addressing the social determinants of health of children and youth: a role for SOPHE members. Health Educ Behav. 2011;38:331–8. 10.1177/1090198111417709 21807954

[89] Bettenhausen JL Winterer CM Colvin JD Health and Poverty of Rural Children: An Under-Researched and Under-Resourced Vulnerable Population. Acad Pediatr. 2021;21:S126–33. 10.1016/j.acap.2021.08.001 34740419

[90] Prictor M Hill S Cochrane Consumers and Communication Review Group leading the field on health communication evidence. J Evid Based Med. 2013;6:216–20. 10.1111/jebm.12066 24325413

[91] Althuis MD Weed DL Frankenfeld CL Evidence-based mapping of design heterogeneity prior to meta-analysis: a systematic review and evidence synthesis. Syst Rev. 2014;3:80. 10.1186/2046-4053-3-80 25055879 PMC4128504

