# Supplementary material for: Treatment-seeking practices of caregivers for children aged less than five years old with diarrhoea in low- and middle-income countries: a systematic review and meta-analysis
Source: J Glob Health. 2025 Apr 25;15:04080. doi: 10.7189/jogh.15.04080 (PMC12023808; doi:10.7189/jogh.15.04080)
Supplement: Online Supplementary Document [file jogh-15-04080-s001.pdf]

Supplement to: Fekadu GA, Wordofa MA, Woldie FB, Fite RO, Alemu K, Worku A, Taddesse L, Bekele D, Tolera G, Chan GJ, Hailemariam D. Treatment-seeking practices of caregivers for children aged less than five years old with diarrhoea in low- and middle-income countries: a systematic review and meta-analysis. J Glob Health. 2025;15:04080.

Table S1: Logic Grid of search and Medical Subject Heading terms for PubMed search forsystematic review and meta-analysis of treatment seeking practices of caregivers for children <5with diarrhea in low- and middle-income countries, 2010–2022.

Condition	Context		Population
Text words	Text words		Text words
• Treatment	• Low-income countries	• Afghanistan	• Under-five children
• Management	• Middle income countries	• Guinea-Bissau	• Child
• Treatment seeking practice	• Developing countries	• Somalia	• Infant
• Care seeking behavior	• Least Developed	• Burkina Faso	• Preschool
• Health care seeking	• Less Developed Countries	• Korea	• Toddler
• Health care utilization	• Third-World Countries	• South Sudan	• Childhood
• Care Seeking Pattern	• Low-Income Countries	• Burundi	• Mother
	• Central African Republic	• Liberia	• Care giver
	• Yemen	• Sudan	
	• Ethiopia	• Madagascar	
	• Niger	• Syria	
	• Gambia	• Chad	
	• Rwanda	• Malawi	
	• Guinea	• Togo	
	• Sierra Leone	• Congo	
	• Angola	• Dem. Rep	
	• Honduras	• Mali	
	• Philippines	• Uganda	
	• Algeria	• Eritrea	
	• India	• Mozambique	
	• Samoa	• Ethiopia	
	• Bangladesh	• Niger	
	• Indonesia	• Gambia	
	• São Tomé	• Rwanda	
	• Principe	• Guinea	
	• Belize	• Sierra Leone	
	• Iran	• Angola	
	• Senegal	• Honduras	
	• Benin	• Philippines	
	• Kenya	• Algeria	
	• Solomon Islands	• India	
	• Bhutan	• Samoa	
	• Kiribati	• Bangladesh	
	• Sri Lanka	• Indonesia	
	• Bolivia	• São Tomé	
	• Kyrgyz Republic	• Principe	
	• Lao PDR	• Tanzania	
	• Tajikistan	• Cabo Verde	
	• Cambodia	• Eswatini	
	• Lesotho	• Nigeria	
	• Timor-Leste	• Zimbabwe	
	• Cameroon	• Ghana	
	• Mauritania	• Pakistan	
	• Tunisia	• Haiti	
	• Comoros	• Papua New Guinea	
	• Micronesia	• Albania	
	• Ukraine	• Gabon	
	• Congo		
	• Mongolia		

	• Uzbekistan<br>• Côte d'Ivoire<br>• Morocco<br>• Vanuatu<br>• Djibouti<br>• Myanmar<br>• Vietnam<br>• Egypt<br>• Nepal<br>• West Bank<br>• Gaza<br>• Brazil<br>• Kazakhstan<br>• South Africa<br>• Bulgaria<br>• Kosovo<br>• St. Lucia<br>• China<br>• Lebanon<br>• St. Vincent<br>• The Grenadines<br>• Colombia<br>• Libya<br>• Suriname<br>• Costa Rica<br>• Malaysia<br>• Dominican Republic<br>• Mauritius<br>• Turkmenistan<br>• Equatorial Guinea<br>• Mexico<br>• Tuvalu<br>• Ecuador<br>• Moldova, Fiji<br>• Montenegro	• Namibia<br>• American Samoa<br>• Georgia<br>• North Macedonia<br>• Argentina<br>• Grenada<br>• Panama<br>• Armenia<br>• Guatemala<br>• Paraguay<br>• El Salvador<br>• Nicaragua<br>• Zambia<br>• Azerbaijan<br>• Guyana<br>• Peru<br>• Belarus<br>• Iraq<br>• Romania<br>• Bosnia<br>• Herzegovina<br>• Jamaica<br>• Russia<br>• Botswana<br>• Jordan<br>• Serbia<br>• Thailand<br>• Cuba<br>• Maldives<br>• Tonga<br>• Dominica<br>• Marshall Islands<br>• Turkey<br>• Equatorial Guinea<br>• Mexico<br>• Tuvalu, Ecuador	
**MeSH terms**<br>• Health care utilization	**MeSH terms**<br>• Low-income countries<br>• Middle income countries<br>• Developing countries<br>• Least Developed Countries<br>• Less Developed Countries<br>• Third-World Countries<br>• Low-Income Countries<br>• Afghanistan<br>• Guinea-Bissau<br>• Somalia<br>• Burkina Faso<br>• Korea<br>• South Sudan<br>• Burundi<br>• Liberia<br>• Sudan<br>• Central African Republic<br>• Madagascar	•<br>• Tunisia<br>• Comoros<br>• Micronesia<br>• Ukraine<br>• Congo<br>• Mongolia<br>• Uzbekistan<br>• Côte d'Ivoire<br>• Morocco<br>• Vanuatu<br>• Djibouti<br>• Myanmar<br>• Vietnam<br>• Egypt<br>• Nepal<br>• West Bank<br>• Gaza<br>• El Salvador<br>• Nicaragua	**MeSH terms**<br>• Child<br>• Infant<br>• Child, preschool<br>• Mothers<br>• Caregivers

	• Syria<br>• Chad<br>• Malawi<br>• Togo<br>• Congo<br>• Dem. Rep<br>• Mali<br>• Uganda<br>• Eritrea<br>• Mozambique<br>• Yemen<br>• Ethiopia<br>• Niger<br>• Gambia<br>• Rwanda<br>• Guinea<br>• Sierra Leone<br>• Angola<br>• Honduras<br>• Philippines<br>• Algeria<br>• India<br>• Samoa<br>• Bangladesh<br>• Indonesia<br>• São Tomé<br>• Principe<br>• Belize<br>• Iran<br>• Senegal<br>• Benin<br>• Kenya<br>• Solomon Islands<br>• Bhutan<br>• Kiribati<br>• Sri Lanka<br>• Bolivia<br>• Kyrgyz Republic<br>• Tanzania<br>• Cabo Verde<br>• Lao PDR<br>• Tajikistan<br>• Cambodia<br>• Lesotho<br>• Timor-Leste<br>• Cameroon<br>• Mauritania<br>• Equatorial Guinea<br>• Mexico<br>• Tuvalu<br>• Ecuador<br>• Moldova, Fiji<br>• Montenegro<br>• Dominican Republic<br>• Mauritius<br>• Turkmenistan	• Zambia<br>• Eswatini<br>• Nigeria<br>• Zimbabwe<br>• Ghana<br>• Pakistan<br>• Haiti<br>• Papua New Guinea<br>• Albania<br>• Gabon<br>• Namibia<br>• American Samoa<br>• Georgia<br>• North Macedonia<br>• Argentina<br>• Grenada<br>• Panama<br>• Armenia<br>• Guatemala<br>• Paraguay<br>• Azerbaijan<br>• Guyana<br>• Peru<br>• Belarus<br>• Iraq<br>• Romania<br>• Bosnia<br>• Herzegovina<br>• Jamaica<br>• Russia<br>• Botswana<br>• Jordan<br>• Serbia<br>• Brazil<br>• Kazakhstan<br>• South Africa<br>• Bulgaria<br>• Kosovo<br>• St. Lucia<br>• China<br>• Lebanon<br>• St. Vincent<br>• The Grenadines<br>• Colombia<br>• Libya<br>• Suriname<br>• Costa Rica<br>• Malaysia<br>• Thailand<br>• Cuba<br>• Maldives<br>• Tonga<br>• Dominica<br>• Marshall Islands<br>• Turkey	

Table S2: Search history results from of PubMed for systematic review and meta-analysis of treatment seeking practices of caregivers for children < 5 years with diarrhea in low andmiddle-income countries, 2010–2022

Search #	Search terms	Search results	Date & time
#5	**((((((Treatment) OR (Management)) OR (Treatment seeking practice)) OR (Care seeking behavior)) OR (Health care seeking)) OR (Health care utilization)) OR (Care Seeking Pattern)) OR (Health care utilization[MeSH Terms])**	6,035,940	February 22, 2022 03:10:52
#6	**(((((((((((Under-five children) OR (Child)) OR (Infant)) OR (Preschool)) OR (Toddler Childhood)) OR (Mother)) OR (Care giver)) OR (Child[MeSH Terms])) OR (Infant[MeSH Terms])) OR (Child, preschool[MeSH Terms])) OR (Mothers[MeSH Terms])) OR (Caregivers[MeSH Terms]))**	1,412,772	February 22, 2022 03:12:23
#2	**((Low-income countries) OR (Middle income countries)) OR (Developing countries)) OR (Least Developed Countries)) OR (Less Developed Countries)) OR (Third-World Countries)) OR (Low-Income Countries)) OR (Central African Republic)) OR (Yemen)) OR (Ethiopia)) OR (Niger)) OR (Gambia)) OR (Rwanda)) OR (Guinea)) OR (Sierra Leone)) OR (Angola)) OR (Honduras)) OR (Philippines)) OR (Algeria)) OR (India)) OR (Samoa)) OR (Bangladesh)) OR (Indonesia)) OR (São Tomé)) OR (Principe)) OR (Belize)) OR (Iran)) OR (Senegal)) OR (Benin)) OR (Kenya)) OR (Solomon Islands)) OR (Bhutan)) OR (Kiribati)) OR (Sri Lanka)) OR (Bolivia)) OR (Kyrgyz Republic)) OR (Lao PDR)) OR (Tajikistan)) OR (Cambodia)) OR (Lesotho)) OR (Timor-Leste)) OR (\)) OR (Cameroon)) OR (Mauritania)) OR (Tunisia)) OR (Comoros)) OR (Micronesia)) OR (Ukraine)) OR (Congo)) OR (Mongolia)) OR (Uzbekistan)) OR (Côte d'Ivoire)) OR (Morocco)) OR (Vanuatu)) OR (Djibouti)) OR (Myanmar)) OR (Vietnam)) OR (Egypt)) OR (Nepal)) OR (West Bank)) OR (Gaza)) OR (Brazil)) OR (Kazakhstan)) OR (South Africa)) OR (Bulgaria)) OR (Kosovo)) OR (St. Lucia)) OR (China)) OR (Lebanon)) OR (St. Vincent)) OR (The Grenadines)) OR (Colombia)) OR (Libya)) OR (Suriname)) OR (Costa Rica)) OR (Malaysia)) OR (Dominican Republic)) OR (Mauritius)) OR (Turkmenistan)) OR (Equatorial Guinea)) OR (Mexico)) OR (Tuvalu)) OR (Ecuador)) OR (Moldova)) OR (Fiji)) OR (Montenegro)) OR (Afghanistan)) OR (Guinea-Bissau)) OR (Somalia)) OR (Burkina Faso)) OR (Korea)) OR (South Sudan)) OR (Burundi)) OR (Liberia)) OR (Sudan)) OR (Madagascar)) OR (Syria)) OR (Chad)) OR (Malawi)) OR (Togo)) OR (Congo)) OR (Dem. Rep)) OR (Mali)) OR (Uganda)) OR (Eritrea)) OR (Mozambique)) OR (Ethiopia)) OR (Niger)) OR (Gambia)) OR (Rwanda)) OR (Guinea Sierra Leone)) OR (Angola)) OR (Honduras)) OR (Philippines)) OR (Algeria)) OR (India)) OR (Samoa)) OR (Bangladesh)) OR (Indonesia)) OR (São Tomé)) OR (Principe)) OR (Tanzania)) OR (Cabo Verde)) OR (Eswatini)) OR (Nigeria)) OR (Zimbabwe)) OR (Ghana)) OR (Pakistan)) OR (Haiti)) OR (Papua New Guinea)) OR (Albania)) OR (Namibia)) OR (American Samoa)) OR (Georgia)) OR (Gabon)) OR (North Macedonia)) OR (Argentina)) OR (Grenada)) OR (Panama)) OR (Armenia)) OR (Guatemala)) OR (Paraguay)) OR (El Salvador)) OR (Nicaragua)) OR (Zambia)) OR (Azerbaijan)) OR (Guyana)) OR (Peru)) OR (Belarus)) OR (Iraq)) OR (Romania)) OR (Bosnia)) OR (Herzegovina)) OR (Jamaica)) OR (Russia)) OR (Botswana)) OR (Jordan)) OR (Serbia)) OR (Thailand)) OR (Cuba)) OR (Maldives)) OR (Tonga)) OR (Dominica)) OR (Marshall Islands)) OR (Turkey)) OR (Equatorial Guinea)) OR (Mexico)) OR (Tuvalu Ecuador)) OR (Low-income countries[MeSH Terms])) OR (Middle income countries[MeSH Terms])) OR (Developing**	4,514,596	February22, 2022 02:25:45

	**countries[MeSH Terms])) OR (Least Developed Countries[MeSH Terms])) OR (Less Developed Countries[MeSH Terms])) OR (Third-World Countries[MeSH Terms])) OR (Low-Income Countries[MeSH Terms])) OR (Afghanistan[MeSH Terms])) OR (Albania[MeSH Terms])) OR (Algeria[MeSH Terms])) OR (Argentina[MeSH Terms])) OR (Armenia[MeSH Terms])) OR (Angola[MeSH Terms])) OR (American Samoa[MeSH Terms])) OR (Azerbaijan[MeSH Terms])) OR (Bangladesh[MeSH Terms])) OR (Belarus[MeSH Terms])) OR (Belize[MeSH Terms])) OR (Benin[MeSH Terms])) OR (Bhutan[MeSH Terms])) OR (Bolivia[MeSH Terms])) OR (Bosnia[MeSH Terms])) OR (Botswana[MeSH Terms])) OR (Brazil[MeSH Terms])) OR (Bulgaria[MeSH Terms])) OR (Burkina Faso[MeSH Terms])) OR (Burundi[MeSH Terms])) OR (Cabo Verde[MeSH Terms])) OR (Cambodia[MeSH Terms])) OR (Cameroon[MeSH Terms])) OR (Central African Republic[MeSH Terms])) OR (Chad[MeSH Terms])) OR (China[MeSH Terms])) OR (Colombia[MeSH Terms])) OR (Comoros[MeSH Terms])) OR (Congo[MeSH Terms])) OR (Congo[MeSH Terms])) OR (Costa Rica[MeSH Terms])) OR (Côte d'Ivoire[MeSH Terms])) OR (Cuba[MeSH Terms])) OR (Dem. Rep[MeSH Terms])) OR (Djibouti[MeSH Terms])) OR (Dominica[MeSH Terms])) OR (Dominican Republic[MeSH Terms])) OR (Ecuador[MeSH Terms])) OR (Egypt[MeSH Terms])) OR (El Salvador[MeSH Terms])) OR (Equatorial Guinea[MeSH Terms])) OR (Eritrea[MeSH Terms])) OR (Eswatini[MeSH Terms])) OR (Ethiopia[MeSH Terms])) OR (Fiji[MeSH Terms])) OR (Gabon[MeSH Terms])) OR (Gambia[MeSH Terms])) OR (Gaza[MeSH Terms])) OR (Georgia[MeSH Terms])) OR (Ghana[MeSH Terms])) OR (Grenada[MeSH Terms])) OR (Guatemala[MeSH Terms])) OR (Guinea[MeSH Terms])) OR (Guinea-Bissau[MeSH Terms])) OR (Guyana[MeSH Terms])) OR (Haiti[MeSH Terms])) OR (Herzegovina[MeSH Terms])) OR (Honduras[MeSH Terms])) OR (India[MeSH Terms])) OR (Indonesia[MeSH Terms])) OR (Iran[MeSH Terms])) OR (Iraq[MeSH Terms])) OR (Jamaica[MeSH Terms])) OR (Jordan[MeSH Terms])) OR (Kazakhstan[MeSH Terms])) OR (Kenya[MeSH Terms])) OR (Kiribati[MeSH Terms])) OR (Korea[MeSH Terms])) OR (Kosovo[MeSH Terms])) OR (Kyrgyz Republic[MeSH Terms])) OR (Lao PDR[MeSH Terms])) OR (Lebanon[MeSH Terms])) OR (Lesotho[MeSH Terms])) OR (Liberia[MeSH Terms])) OR (Libya[MeSH Terms])) OR (Madagascar[MeSH Terms])) OR (Malawi[MeSH Terms])) OR (Malaysia[MeSH Terms])) OR (Maldives[MeSH Terms])) OR (Mali[MeSH Terms])) OR (Marshall Islands[MeSH Terms])) OR (Mauritania[MeSH Terms])) OR (Mauritius[MeSH Terms])) OR (Mexico[MeSH Terms])) OR (Micronesia[MeSH Terms])) OR (Moldova[MeSH Terms])) OR (Mongolia[MeSH Terms])) OR (Montenegro[MeSH Terms])) OR (morocco[MeSH Terms])) OR (Mozambique[MeSH Terms])) OR (Myanmar[MeSH Terms])) OR (Namibia[MeSH Terms])) OR (Nepal[MeSH Terms])) OR (Nicaragua[MeSH Terms])) OR (Niger[MeSH Terms])) OR (Nigeria[MeSH Terms])) OR (North Macedonia[MeSH Terms])) OR (Pakistan[MeSH Terms])) OR (Panama[MeSH Terms])) OR (Papua New Guinea[MeSH Terms])) OR (Paraguay[MeSH Terms])) OR (Peru[MeSH Terms])) OR (Philippines[MeSH Terms])) OR (Principe[MeSH Terms])) OR (Romania[MeSH Terms])) OR (Russia[MeSH Terms])) OR (Rwanda[MeSH Terms])) OR (Samoa[MeSH Terms])) OR (São Tomé[MeSH Terms])) OR (Senegal[MeSH Terms])) OR (Serbia[MeSH Terms])) OR (Sierra Leone[MeSH Terms])) OR (Solomon Islands[MeSH Terms])) OR (Somalia[MeSH Terms])) OR (South Africa[MeSH Terms])) OR (South Sudan[MeSH Terms])) OR (St. Lucia[MeSH Terms])) OR (St. Vincent[MeSH Terms])) OR (Sudan[MeSH Terms])) OR (Suriname[MeSH Terms])) OR (Sri Lanka[MeSH Terms])) OR (Syria[MeSH Terms])) OR (Tajikistan[MeSH Terms])) OR (Tanzania[MeSH Terms])) OR (Thailand[MeSH Terms])) OR (The Grenadines[MeSH Terms])) OR (Timor-Leste[MeSH Terms])) OR (Togo[MeSH Terms])) OR (Tonga[MeSH Terms])) OR (Tunisia[MeSH Terms])) OR (Turkey[MeSH Terms])) OR (Turkmenistan[MeSH Terms])) OR (Tuvalu[MeSH Terms])) OR (Uganda[MeSH Terms])) OR (Ukraine[MeSH**		

	**Terms])) OR (Uzbekistan[MeSH Terms])) OR (Vanuatu[MeSH Terms])) OR (Vietnam[MeSH Terms])) OR (West Bank[MeSH Terms])) OR (Yemen[MeSH Terms])) OR (Zambia[MeSH Terms])) OR (Zimbabwe[MeSH Terms])**		
#7 **((#2) AND (#5)) AND (#6)**	((("poverty"[MeSH Terms] OR "poverty"[All Fields] OR ("low"[All Fields] AND "income"[All Fields]) OR "low income"[All Fields]) AND ("countries"[All Fields] OR "country"[All Fields] OR "country s"[All Fields] OR "countrys"[All Fields])) OR (("middle"[All Fields] OR "middles"[All Fields]) AND ("income"[MeSH Terms] OR "income"[All Fields] OR "incomes"[All Fields] OR "income s"[All Fields]) AND ("countries"[All Fields] OR "country"[All Fields] OR "country s"[All Fields] OR "countrys"[All Fields])) OR ("developing countries"[MeSH Terms] OR ("developing"[All Fields] AND "countries"[All Fields]) OR "developing countries"[All Fields]) OR ("developing countries"[MeSH Terms] OR ("developing"[All Fields] AND "countries"[All Fields]) OR "developing countries"[All Fields] OR ("least"[All Fields] AND "developed"[All Fields] AND "countries"[All Fields]) OR "least developed countries"[All Fields]) OR ("developing countries"[MeSH Terms] OR ("developing"[All Fields] AND "countries"[All Fields]) OR "developing countries"[All Fields] OR ("less"[All Fields] AND "developed"[All Fields] AND "countries"[All Fields]) OR "less developed countries"[All Fields]) OR ("developing countries"[MeSH Terms] OR ("developing"[All Fields] AND "countries"[All Fields]) OR "developing countries"[All Fields] OR ("third"[All Fields] AND "world"[All Fields] AND "countries"[All Fields]) OR "third world countries"[All Fields]) OR (("poverty"[MeSH Terms] OR "poverty"[All Fields] OR ("low"[All Fields] AND "income"[All Fields]) OR "low income"[All Fields]) AND ("countries"[All Fields] OR "country"[All Fields] OR "country s"[All Fields] OR "countrys"[All Fields])) OR ("central african republic"[MeSH Terms] OR ("central"[All Fields] AND "african"[All Fields] AND "republic"[All Fields]) OR "central african republic"[All Fields]) OR ("yemen"[MeSH Terms] OR "yemen"[All Fields]) OR ("ethiopia"[MeSH Terms] OR "ethiopia"[All Fields] OR "ethiopia s"[All Fields]) OR ("niger"[MeSH Terms] OR "niger"[All Fields]) OR ("gambia"[MeSH Terms] OR "gambia"[All Fields] OR "gambia s"[All Fields]) OR ("rwanda"[MeSH Terms] OR "rwanda"[All Fields] OR "rwanda s"[All Fields]) OR ("guinea"[MeSH Terms] OR "guinea"[All Fields] OR "guinea s"[All Fields] OR "guineas"[All Fields]) OR ("sierra leone"[MeSH Terms] OR ("sierra"[All Fields] AND "leone"[All Fields]) OR "sierra leone"[All Fields]) OR ("angola"[MeSH Terms] OR "angola"[All Fields] OR "angola s"[All Fields]) OR ("honduras"[MeSH Terms] OR "honduras"[All Fields]) OR ("philippine"[All Fields] OR "philippines"[MeSH Terms] OR "philippines"[All Fields]) OR ("algeria"[MeSH Terms] OR "algeria"[All Fields]) OR ("india"[MeSH Terms] OR "india"[All Fields] OR "india s"[All Fields] OR "indias"[All Fields]) OR ("samoa"[MeSH Terms] OR "samoa"[All Fields] OR "samoas"[All Fields]) OR ("bangladesh"[MeSH Terms] OR "bangladesh"[All Fields] OR "bangladesh s"[All Fields]) OR ("indonesia"[MeSH Terms] OR "indonesia"[All Fields] OR "indonesia s"[All Fields] OR "indonesias"[All Fields]) OR ("sao"[All Fields] AND "tome"[All Fields]) OR ("principe"[All Fields] OR "principes"[All Fields]) OR ("belize"[MeSH Terms] OR "belize"[All Fields]) OR ("iran"[MeSH Terms] OR "iran"[All Fields]) OR ("senegal"[MeSH Terms] OR "senegal"[All Fields] OR "senegal s"[All Fields]) OR ("benin"[MeSH Terms] OR "benin"[All Fields] OR "benin s"[All Fields]) OR ("kenya"[MeSH Terms] OR "kenya"[All Fields] OR "kenya s"[All Fields]) OR ("melanesia"[MeSH Terms] OR "melanesia"[All Fields] OR ("solomon"[All Fields] AND "islands"[All Fields]) OR "solomon islands"[All Fields]) OR ("bhutan"[MeSH Terms] OR "bhutan"[All Fields] OR "bhutan s"[All Fields]) OR ("micronesia"[MeSH Terms] OR "micronesia"[All Fields] OR "kiribati"[All Fields]) OR ("sri lanka"[MeSH Terms] OR ("sri"[All Fields] AND "lanka"[All Fields]) OR "sri lanka"[All Fields]) OR ("bolivia"[MeSH Terms] OR "bolivia"[All Fields]) OR ("kyrgyzstan"[MeSH Terms] OR "kyrgyzstan"[All Fields] OR ("kyrgyz"[All Fields] AND "republic"[All Fields]) OR "kyrgyz republic"[All Fields]) OR ("lao"[All Fields] AND "pdr"[All Fields]) OR ("tajikistan"[MeSH Terms] OR "tajikistan"[All Fields]) OR ("cambodia"[MeSH Terms] OR "cambodia"[All Fields] OR "cambodia s"[All Fields]) OR ("lesotho"[MeSH Terms] OR "lesotho"[All Fields]) OR ("timor leste"[MeSH Terms] OR "timor leste"[All Fields] OR ("timor"[All Fields] AND "leste"[All	248,300	February 22, 2022<br><br>03:15:53

	Fields]) OR "timor leste"[All Fields]) OR ("cameroon"[MeSH Terms] OR "cameroon"[All Fields] OR "cameroons"[All Fields] OR "cameroon s"[All Fields]) OR ("mauritania"[MeSH Terms] OR "mauritania"[All Fields]) OR ("tunisia"[MeSH Terms] OR "tunisia"[All Fields]) OR ("comoros"[MeSH Terms] OR "comoros"[All Fields] OR "comoro"[All Fields]) OR ("micronesia"[MeSH Terms] OR "micronesia"[All Fields]) OR ("ukraine"[MeSH Terms] OR "ukraine"[All Fields] OR "ukraine s"[All Fields]) OR ("congo"[MeSH Terms] OR "congo"[All Fields]) OR ("mongolia"[MeSH Terms] OR "mongolia"[All Fields] OR "mongolia s"[All Fields]) OR ("uzbekistan"[MeSH Terms] OR "uzbekistan"[All Fields]) OR ("cote d ivoire"[MeSH Terms] OR ("cote"[All Fields] AND "d ivoire"[All Fields]) OR "cote d ivoire"[All Fields]) OR ("morocco"[MeSH Terms] OR "morocco"[All Fields]) OR ("vanuatu"[MeSH Terms] OR "vanuatu"[All Fields]) OR ("djibouti"[MeSH Terms] OR "djibouti"[All Fields]) OR ("myanmar"[MeSH Terms] OR "myanmar"[All Fields] OR "myanmar s"[All Fields] OR "myanmars"[All Fields]) OR ("vietnam"[MeSH Terms] OR "vietnam"[All Fields] OR "vietnam s"[All Fields]) OR ("egypt"[MeSH Terms] OR "egypt"[All Fields] OR "egypt s"[All Fields]) OR ("nepal"[MeSH Terms] OR "nepal"[All Fields] OR "nepal s"[All Fields]) OR ("middle east"[MeSH Terms] OR ("middle"[All Fields] AND "east"[All Fields]) OR "middle east"[All Fields] OR ("west"[All Fields] AND "bank"[All Fields]) OR "west bank"[All Fields]) OR "gaza"[All Fields] OR ("brazil"[MeSH Terms] OR "brazil"[All Fields] OR "brazil s"[All Fields] OR "brazils"[All Fields]) OR ("kazakhstan"[MeSH Terms] OR "kazakhstan"[All Fields] OR "kazakhstan s"[All Fields]) OR ("south africa"[MeSH Terms] OR ("south"[All Fields] AND "africa"[All Fields]) OR "south africa"[All Fields]) OR ("bulgaria"[MeSH Terms] OR "bulgaria"[All Fields]) OR ("kosovo"[MeSH Terms] OR "kosovo"[All Fields] OR "kosovo s"[All Fields]) OR ("saint lucia"[MeSH Terms] OR ("saint"[All Fields] AND "lucia"[All Fields]) OR "saint lucia"[All Fields] OR ("st"[All Fields] AND "lucia"[All Fields]) OR "st lucia"[All Fields]) OR ("china"[MeSH Terms] OR "china"[All Fields] OR "china s"[All Fields] OR "chinas"[All Fields]) OR ("lebanon"[MeSH Terms] OR "lebanon"[All Fields] OR "lebanon s"[All Fields]) OR (vincent, st[Investigator] OR st vincent[Author] OR st vincent[Investigator]) OR ("saint vincent and the grenadines"[MeSH Terms] OR ("saint"[All Fields] AND "vincent"[All Fields] AND "grenadines"[All Fields]) OR "saint vincent and the grenadines"[All Fields] OR "grenadines"[All Fields]) OR ("colombia"[MeSH Terms] OR "colombia"[All Fields] OR "colombia s"[All Fields]) OR ("libya"[MeSH Terms] OR "libya"[All Fields]) OR ("suriname"[MeSH Terms] OR "suriname"[All Fields] OR "surinam"[All Fields]) OR ("costa rica"[MeSH Terms] OR ("costa"[All Fields] AND "rica"[All Fields]) OR "costa rica"[All Fields]) OR ("malaysia"[MeSH Terms] OR "malaysia"[All Fields] OR "malaysia s"[All Fields]) OR ("dominican republic"[MeSH Terms] OR ("dominican"[All Fields] AND "republic"[All Fields]) OR "dominican republic"[All Fields]) OR ("mauritius"[MeSH Terms] OR "mauritius"[All Fields]) OR ("turkmenistan"[MeSH Terms] OR "turkmenistan"[All Fields]) OR ("equatorial guinea"[MeSH Terms] OR ("equatorial"[All Fields] AND "guinea"[All Fields]) OR "equatorial guinea"[All Fields]) OR ("mexico"[MeSH Terms] OR "mexico"[All Fields] OR "mexico s"[All Fields] OR "mexicos"[All Fields]) OR ("micronesia"[MeSH Terms] OR "micronesia"[All Fields] OR "tuvalu"[All Fields]) OR ("ecuador"[MeSH Terms] OR "ecuador"[All Fields] OR "ecuador s"[All Fields]) OR ("moldova"[MeSH Terms] OR "moldova"[All Fields]) OR ("fiji"[MeSH Terms] OR "fiji"[All Fields]) OR ("montenegro"[MeSH Terms] OR "montenegro"[All Fields]) OR ("afghanistan"[MeSH Terms] OR "afghanistan"[All Fields] OR "afghanistan s"[All Fields]) OR ("guinea bissau"[MeSH Terms] OR "guinea bissau"[All Fields] OR ("guinea"[All Fields] AND "bissau"[All Fields]) OR "guinea bissau"[All Fields]) OR ("somalia"[MeSH Terms] OR "somalia"[All Fields]) OR ("burkina faso"[MeSH Terms] OR ("burkina"[All Fields] AND "faso"[All Fields]) OR "burkina faso"[All Fields]) OR ("korea"[MeSH Terms] OR "korea"[All Fields] OR "korea s"[All Fields] OR "koreas"[All Fields]) OR ("south sudan"[MeSH Terms] OR ("south"[All Fields] AND "sudan"[All Fields]) OR "south sudan"[All Fields]) OR ("burundi"[MeSH Terms] OR "burundi"[All Fields]) OR ("liberia"[MeSH Terms] OR "liberia"[All Fields] OR "liberia s"[All Fields]) OR ("sudan"[MeSH Terms] OR "sudan"[All Fields] OR "sudans"[All Fields] OR "sudan s"[All Fields]) OR ("madagascar"[MeSH Terms] OR		

	"madagascar"[All Fields] OR "madagascar s"[All Fields]) OR ("syria"[MeSH Terms] OR "syria"[All Fields] OR "syria s"[All Fields]) OR ("chad"[MeSH Terms] OR "chad"[All Fields]) OR ("malawi"[MeSH Terms] OR "malawi"[All Fields] OR "malawi s"[All Fields]) OR ("togo"[MeSH Terms] OR "togo"[All Fields]) OR ("congo"[MeSH Terms] OR "congo"[All Fields]) OR ("dem"[All Fields] AND "rep"[All Fields]) OR ("mali"[MeSH Terms] OR "mali"[All Fields]) OR ("uganda"[MeSH Terms] OR "uganda"[All Fields] OR "uganda s"[All Fields]) OR ("eritrea"[MeSH Terms] OR "eritrea"[All Fields]) OR ("mozambique"[MeSH Terms] OR "mozambique"[All Fields] OR "mozambique s"[All Fields]) OR ("ethiopia"[MeSH Terms] OR "ethiopia"[All Fields] OR "ethiopia s"[All Fields]) OR ("niger"[MeSH Terms] OR "niger"[All Fields]) OR ("gambia"[MeSH Terms] OR "gambia"[All Fields] OR "gambia s"[All Fields]) OR ("rwanda"[MeSH Terms] OR "rwanda"[All Fields] OR "rwanda s"[All Fields]) OR (("guinea"[MeSH Terms] OR "guinea"[All Fields] OR "guinea s"[All Fields] OR "guineas"[All Fields]) AND ("sierra leone"[MeSH Terms] OR ("sierra"[All Fields] AND "leone"[All Fields]) OR "sierra leone"[All Fields])) OR ("angola"[MeSH Terms] OR "angola"[All Fields] OR "angola s"[All Fields]) OR ("honduras"[MeSH Terms] OR "honduras"[All Fields]) OR ("philippine"[All Fields] OR "philippines"[MeSH Terms] OR "philippines"[All Fields]) OR ("algeria"[MeSH Terms] OR "algeria"[All Fields]) OR ("india"[MeSH Terms] OR "india"[All Fields] OR "india s"[All Fields] OR "indias"[All Fields]) OR ("samoa"[MeSH Terms] OR "samoa"[All Fields] OR "samoas"[All Fields]) OR ("bangladesh"[MeSH Terms] OR "bangladesh"[All Fields] OR "bangladesh s"[All Fields]) OR ("indonesia"[MeSH Terms] OR "indonesia"[All Fields] OR "indonesia s"[All Fields] OR "indonesias"[All Fields]) OR ("sao"[All Fields] AND "tome"[All Fields]) OR ("principe"[All Fields] OR "principes"[All Fields]) OR ("tanzania"[MeSH Terms] OR "tanzania"[All Fields] OR "tanzania s"[All Fields]) OR ("cabo verde"[MeSH Terms] OR ("cabo"[All Fields] AND "verde"[All Fields]) OR "cabo verde"[All Fields]) OR ("eswatini"[MeSH Terms] OR "eswatini"[All Fields]) OR ("nigeria"[MeSH Terms] OR "nigeria"[All Fields] OR "nigeria s"[All Fields]) OR ("zimbabwe"[MeSH Terms] OR "zimbabwe"[All Fields] OR "zimbabwe s"[All Fields]) OR ("ghana"[MeSH Terms] OR "ghana"[All Fields] OR "ghana s"[All Fields]) OR ("pakistan"[MeSH Terms] OR "pakistan"[All Fields] OR "pakistan s"[All Fields]) OR ("haiti"[MeSH Terms] OR "haiti"[All Fields] OR "haiti s"[All Fields]) OR ("papua new guinea"[MeSH Terms] OR ("papua"[All Fields] AND "new"[All Fields] AND "guinea"[All Fields]) OR "papua new guinea"[All Fields]) OR ("albania"[MeSH Terms] OR "albania"[All Fields]) OR ("namibia"[MeSH Terms] OR "namibia"[All Fields]) OR ("american samoa"[MeSH Terms] OR ("american"[All Fields] AND "samoa"[All Fields]) OR "american samoa"[All Fields]) OR ("georgia"[MeSH Terms] OR "georgia"[All Fields] OR "georgia republic"[MeSH Terms] OR ("georgia"[All Fields] AND "republic"[All Fields]) OR "georgia republic"[All Fields] OR "georgia s"[All Fields]) OR ("gabon"[MeSH Terms] OR "gabon"[All Fields]) OR ("republic of north macedonia"[MeSH Terms] OR ("republic"[All Fields] AND "north"[All Fields] AND "macedonia"[All Fields]) OR "republic of north macedonia"[All Fields] OR ("north"[All Fields] AND "macedonia"[All Fields]) OR "north macedonia"[All Fields]) OR ("argentina"[MeSH Terms] OR "argentina"[All Fields] OR "argentina s"[All Fields] OR "argentinae"[All Fields]) OR ("grenada"[MeSH Terms] OR "grenada"[All Fields]) OR ("panama"[MeSH Terms] OR "panama"[All Fields] OR "panama s"[All Fields]) OR ("armenia"[MeSH Terms] OR "armenia"[All Fields]) OR ("guatemala"[MeSH Terms] OR "guatemala"[All Fields] OR "guatemala s"[All Fields]) OR ("paraguai"[All Fields] OR "paraguay"[MeSH Terms] OR "paraguay"[All Fields]) OR ("el salvador"[MeSH Terms] OR ("el"[All Fields] AND "salvador"[All Fields]) OR "el salvador"[All Fields]) OR ("nicaragua"[MeSH Terms] OR "nicaragua"[All Fields] OR "nicaragua s"[All Fields]) OR ("zambia"[MeSH Terms] OR "zambia"[All Fields] OR "zambia s"[All Fields]) OR ("azerbaijan"[MeSH Terms] OR "azerbaijan"[All Fields]) OR ("guyana"[MeSH Terms] OR "guyana"[All Fields]) OR ("peru"[MeSH Terms] OR "peru"[All Fields]) OR ("republic of belarus"[MeSH Terms] OR ("republic"[All Fields] AND "belarus"[All Fields]) OR "republic of belarus"[All Fields] OR "belarus"[All Fields]) OR ("iraq"[MeSH Terms] OR "iraq"[All Fields]) OR ("romania"[MeSH Terms] OR "romania"[All Fields] OR "romania s"[All Fields]) OR		

	("bosnia and herzegovina"[MeSH Terms] OR ("bosnia"[All Fields] AND "herzegovina"[All Fields]) OR "bosnia and herzegovina"[All Fields] OR "bosnia"[All Fields]) OR ("bosnia and herzegovina"[MeSH Terms] OR ("bosnia"[All Fields] AND "herzegovina"[All Fields]) OR "bosnia and herzegovina"[All Fields] OR "herzegovina"[All Fields]) OR ("jamaica"[MeSH Terms] OR "jamaica"[All Fields] OR "jamaica s"[All Fields]) OR ("russia"[MeSH Terms] OR "russia"[All Fields] OR "russia s"[All Fields] OR "russias"[All Fields]) OR ("botswana"[MeSH Terms] OR "botswana"[All Fields] OR "botswana s"[All Fields]) OR ("jordan"[MeSH Terms] OR "jordan"[All Fields]) OR ("serbia"[MeSH Terms] OR "serbia"[All Fields]) OR ("thailand"[MeSH Terms] OR "thailand"[All Fields] OR "thailand s"[All Fields]) OR ("cuba"[MeSH Terms] OR "cuba"[All Fields]) OR ("indian ocean islands"[MeSH Terms] OR ("indian"[All Fields] AND "ocean"[All Fields] AND "islands"[All Fields]) OR "indian ocean islands"[All Fields] OR "maldives"[All Fields] OR "maldive"[All Fields]) OR ("tonga"[MeSH Terms] OR "tonga"[All Fields] OR "tonga s"[All Fields]) OR ("dominica"[MeSH Terms] OR "dominica"[All Fields]) OR ("micronesia"[MeSH Terms] OR "micronesia"[All Fields] OR ("marshall"[All Fields] AND "islands"[All Fields]) OR "marshall islands"[All Fields]) OR ("turkey"[MeSH Terms] OR "turkey"[All Fields] OR "turkey s"[All Fields] OR "turkeys"[MeSH Terms] OR "turkeys"[All Fields]) OR ("equatorial guinea"[MeSH Terms] OR ("equatorial"[All Fields] AND "guinea"[All Fields]) OR "equatorial guinea"[All Fields]) OR ("mexico"[MeSH Terms] OR "mexico"[All Fields] OR "mexico s"[All Fields] OR "mexicos"[All Fields]) OR (("micronesia"[MeSH Terms] OR "micronesia"[All Fields] OR "tuvalu"[All Fields]) AND ("ecuador"[MeSH Terms] OR "ecuador"[All Fields] OR "ecuador s"[All Fields])) OR "developing countries"[MeSH Terms] OR "developing countries"[MeSH Terms] OR "developing countries"[MeSH Terms] OR "developing countries"[MeSH Terms] OR "afghanistan"[MeSH Terms] OR "albania"[MeSH Terms] OR "algeria"[MeSH Terms] OR "argentina"[MeSH Terms] OR "armenia"[MeSH Terms] OR "angola"[MeSH Terms] OR "american samoa"[MeSH Terms] OR "azerbaijan"[MeSH Terms] OR "bangladesh"[MeSH Terms] OR "republic of belarus"[MeSH Terms] OR "belize"[MeSH Terms] OR "benin"[MeSH Terms] OR "bhutan"[MeSH Terms] OR "bolivia"[MeSH Terms] OR "bosnia and herzegovina"[MeSH Terms] OR "botswana"[MeSH Terms] OR "brazil"[MeSH Terms] OR "bulgaria"[MeSH Terms] OR "burkina faso"[MeSH Terms] OR "burundi"[MeSH Terms] OR "cabo verde"[MeSH Terms] OR "cambodia"[MeSH Terms] OR "cameroon"[MeSH Terms] OR "central african republic"[MeSH Terms] OR "chad"[MeSH Terms] OR "china"[MeSH Terms] OR "colombia"[MeSH Terms] OR "comoros"[MeSH Terms] OR "congo"[MeSH Terms] OR "congo"[MeSH Terms] OR "costa rica"[MeSH Terms] OR "cote d ivoire"[MeSH Terms] OR "cuba"[MeSH Terms] OR "djibouti"[MeSH Terms] OR "dominica"[MeSH Terms] OR "dominican republic"[MeSH Terms] OR "ecuador"[MeSH Terms] OR "egypt"[MeSH Terms] OR "el salvador"[MeSH Terms] OR "equatorial guinea"[MeSH Terms] OR "eritrea"[MeSH Terms] OR "eswatini"[MeSH Terms] OR "ethiopia"[MeSH Terms] OR "fiji"[MeSH Terms] OR "gabon"[MeSH Terms] OR "gambia"[MeSH Terms] OR ("georgia"[MeSH Terms] OR "georgia republic"[MeSH Terms]) OR "ghana"[MeSH Terms] OR "grenada"[MeSH Terms] OR "guatemala"[MeSH Terms] OR "guinea"[MeSH Terms] OR "guinea bissau"[MeSH Terms] OR "guyana"[MeSH Terms] OR "haiti"[MeSH Terms] OR "bosnia and herzegovina"[MeSH Terms] OR "honduras"[MeSH Terms] OR "india"[MeSH Terms] OR "indonesia"[MeSH Terms] OR "iran"[MeSH Terms] OR "iraq"[MeSH Terms] OR "jamaica"[MeSH Terms] OR "jordan"[MeSH Terms] OR "kazakhstan"[MeSH Terms] OR "kenya"[MeSH Terms] OR "micronesia"[MeSH Terms] OR "korea"[MeSH Terms] OR "kosovo"[MeSH Terms] OR "kyrgyzstan"[MeSH Terms] OR "lebanon"[MeSH Terms] OR "lesotho"[MeSH Terms] OR "liberia"[MeSH Terms] OR "libya"[MeSH Terms] OR "madagascar"[MeSH Terms] OR "malawi"[MeSH Terms] OR "malaysia"[MeSH Terms] OR "indian ocean islands"[MeSH Terms] OR "mali"[MeSH Terms] OR "micronesia"[MeSH Terms] OR "mauritania"[MeSH Terms] OR "mauritius"[MeSH Terms] OR "mexico"[MeSH Terms] OR "micronesia"[MeSH Terms] OR "moldova"[MeSH Terms] OR "mongolia"[MeSH Terms] OR "montenegro"[MeSH Terms] OR "morocco"[MeSH Terms] OR "mozambique"[MeSH Terms] OR "myanmar"[MeSH Terms]		

	OR "namibia"[MeSH Terms] OR "nepal"[MeSH Terms] OR "nicaragua"[MeSH Terms] OR "niger"[MeSH Terms] OR "nigeria"[MeSH Terms] OR "republic of north macedonia"[MeSH Terms] OR "pakistan"[MeSH Terms] OR "panama"[MeSH Terms] OR "papua new guinea"[MeSH Terms] OR "paraguay"[MeSH Terms] OR "peru"[MeSH Terms] OR "philippines"[MeSH Terms] OR "romania"[MeSH Terms] OR "russia"[MeSH Terms] OR "rwanda"[MeSH Terms] OR "samoa"[MeSH Terms] OR "senegal"[MeSH Terms] OR "serbia"[MeSH Terms] OR "sierra leone"[MeSH Terms] OR "melanesia"[MeSH Terms] OR "somalia"[MeSH Terms] OR "south africa"[MeSH Terms] OR "south sudan"[MeSH Terms] OR "saint lucia"[MeSH Terms] OR "sudan"[MeSH Terms] OR "suriname"[MeSH Terms] OR "sri lanka"[MeSH Terms] OR "syria"[MeSH Terms] OR "tajikistan"[MeSH Terms] OR "tanzania"[MeSH Terms] OR "thailand"[MeSH Terms] OR ("saint vincent and the grenadines"[MeSH Terms]) OR "timor leste"[MeSH Terms] OR "togo"[MeSH Terms] OR "tonga"[MeSH Terms] OR "tunisia"[MeSH Terms] OR "turkey"[MeSH Terms] OR "turkmenistan"[MeSH Terms] OR "micronesia"[MeSH Terms] OR "uganda"[MeSH Terms] OR "ukraine"[MeSH Terms] OR "uzbekistan"[MeSH Terms] OR "vanuatu"[MeSH Terms] OR "vietnam"[MeSH Terms] OR "middle east"[MeSH Terms] OR "yemen"[MeSH Terms] OR "zambia"[MeSH Terms] OR "zimbabwe"[MeSH Terms]) AND ("therapeutics"[MeSH Terms] OR "therapeutics"[All Fields] OR "treatments"[All Fields] OR "therapy"[MeSH Subheading] OR "therapy"[All Fields] OR "treatment"[All Fields] OR "treatment s"[All Fields] OR ("manage"[All Fields] OR "managed"[All Fields] OR "management s"[All Fields] OR "managements"[All Fields] OR "manager"[All Fields] OR "manager s"[All Fields] OR "managers"[All Fields] OR "manages"[All Fields] OR "managing"[All Fields] OR "managment"[All Fields] OR "organization and administration"[MeSH Terms] OR ("organization"[All Fields] AND "administration"[All Fields]) OR "organization and administration"[All Fields] OR "management"[All Fields] OR "disease management"[MeSH Terms] OR ("disease"[All Fields] AND "management"[All Fields]) OR "disease management"[All Fields]) OR (("therapeutics"[MeSH Terms] OR "therapeutics"[All Fields] OR "treatments"[All Fields] OR "therapy"[MeSH Subheading] OR "therapy"[All Fields] OR "treatment"[All Fields] OR "treatment s"[All Fields]) AND ("seeking"[All Fields] OR "seeks"[All Fields]) AND ("practicability"[All Fields] OR "practicable"[All Fields] OR "practical"[All Fields] OR "practicalities"[All Fields] OR "practicality"[All Fields] OR "practically"[All Fields] OR "practicals"[All Fields] OR "practice"[All Fields] OR "practice s"[All Fields] OR "practiced"[All Fields] OR "practices"[All Fields] OR "practicing"[All Fields])) OR ("care"[All Fields] AND ("seeking"[All Fields] OR "seeks"[All Fields]) AND ("behavior"[MeSH Terms] OR "behavior"[All Fields] OR "behavioral"[All Fields] OR "behavioural"[All Fields] OR "behavior s"[All Fields] OR "behaviorally"[All Fields] OR "behaviour"[All Fields] OR "behaviourally"[All Fields] OR "behaviours"[All Fields] OR "behaviors"[All Fields] OR "pattern"[All Fields] OR "pattern s"[All Fields] OR "patternability"[All Fields] OR "patternable"[All Fields] OR "patterned"[All Fields] OR "patterning"[All Fields] OR "patternings"[All Fields] OR "patterns"[All Fields])) OR (("delivery of health care"[MeSH Terms] OR ("delivery"[All Fields] AND "health"[All Fields] AND "care"[All Fields]) OR "delivery of health care"[All Fields] OR ("health"[All Fields] AND "care"[All Fields]) OR "health care"[All Fields]) AND ("seeking"[All Fields] OR "seeks"[All Fields])) OR ("patient acceptance of health care"[MeSH Terms] OR ("patient"[All Fields] AND "acceptance"[All Fields] AND "health"[All Fields] AND "care"[All Fields]) OR "patient acceptance of health care"[All Fields] OR ("health"[All Fields] AND "care"[All Fields] AND "utilization"[All Fields]) OR "health care utilization"[All Fields]) OR ("care"[All Fields] AND ("seeking"[All Fields] OR "seeks"[All Fields]) AND ("behavior"[MeSH Terms] OR "behavior"[All Fields] OR "behavioral"[All Fields] OR "behavioural"[All Fields] OR "behavior s"[All Fields] OR "behaviorally"[All Fields] OR "behaviour"[All Fields] OR "behaviourally"[All Fields] OR "behaviours"[All Fields] OR "behaviors"[All Fields] OR "pattern"[All Fields] OR "pattern s"[All Fields] OR "patternability"[All Fields] OR "patternable"[All Fields] OR "patterned"[All Fields] OR "patterning"[All Fields] OR "patternings"[All Fields] OR "patterns"[All Fields])) OR "patient acceptance of health care"[MeSH Terms]) AND		

	(("under five"[All Fields] AND ("child"[MeSH Terms] OR "child"[All Fields] OR "children"[All Fields] OR "child s"[All Fields] OR "children s"[All Fields] OR "childrens"[All Fields] OR "childs"[All Fields])) OR ("child"[MeSH Terms] OR "child"[All Fields] OR "children"[All Fields] OR "child s"[All Fields] OR "children s"[All Fields] OR "childrens"[All Fields] OR "childs"[All Fields]) OR ("infant"[MeSH Terms] OR "infant"[All Fields] OR "infants"[All Fields] OR "infant s"[All Fields]) OR ("child, preschool"[MeSH Terms] OR ("child"[All Fields] AND "preschool"[All Fields]) OR "preschool child"[All Fields] OR "preschooler"[All Fields] OR "preschoolers"[All Fields] OR "preschool"[All Fields] OR "preschooler s"[All Fields] OR "preschools"[All Fields]) OR (("toddler"[All Fields] OR "toddler s"[All Fields] OR "toddlers"[All Fields]) AND ("childhood"[All Fields] OR "childhoods"[All Fields])) OR ("mother s"[All Fields] OR "mothered"[All Fields] OR "mothers"[MeSH Terms] OR "mothers"[All Fields] OR "mother"[All Fields] OR "mothering"[All Fields]) OR ("caregivers"[MeSH Terms] OR "caregivers"[All Fields] OR ("care"[All Fields] AND "giver"[All Fields]) OR "care giver"[All Fields]) OR "child"[MeSH Terms] OR "infant"[MeSH Terms] OR "child, preschool"[MeSH Terms] OR "mothers"[MeSH Terms] OR "caregivers"[MeSH Terms])		

Table S3: Characteristics of studies included in the systematic review and meta-analysis of treatment seeking practices of caregivers for children < 5 years with diarrhea in low and middle-income countries, 2010–2022.

Author (reference)	Publication Status	Study Setting	Study Country	World Bank category	Study Period	Residence	Study Design	Sample	Prevalence	Sampling Method	Methodology
Ahmed S et al., 2020 (21)	Published	Population	Bangladesh	Lower middle	2019	Urban and rural	Cross-sectional	1598	28.00	Stratified cluster	Quantitative
Diaz T et al., 2013 (68)	Published	Populationca	Sierra Leone	Lower middle	June and July 2010 quantitative and April 2010 and later in July 2010 Qualitative	Rural	Cross-sectional	5951	87.00	Random	Both qualitative and quantitative
Aggarwal AK et al., 2018 (44)	Published	Population	India	Lower middle	October 2013 and April 2014	Urban and rural	Cross-sectional	262	7.6	Random	Quantitative
Fissehaye T et al., 2018 (31)	Published	Population	Ethiopia	Low	March 2015 to June, 2016	Urban	Cross-sectional		72.5	Systematic	Quantitative
Bruce N et al., 2014 (43)	Published	Population	Guatemala	Upper middle	2 surveys from October 2008 to May 2009, and June to December 2009	Rural	Cross-sectional	481	30.6	Cannot retrieve	Quantitative
Gao W et al., 2012 (27)	Published	Population	China	Upper middle	June - August 2005	Semi urban	Cross-sectional	1040	76.54	Random	Quantitative
Hamooya BM et al., 2020 (74)	Published	Population	Zambia	Low	July 2006 - June 2007	Rural	Cross-sectional	1212	32	Systematic	Quantitative
Quadri F et al., 2013 (67)	Published	Population	Pakistan	Lower middle	April 2009 - March 2011	Semi urban	Cross-sectional	349	80.3	Random	Quantitative
Sarker AR et al., 2016 (22)	Published	Population	Bangladesh	Lower middle	June 28, 2014 - November 9, 2014	Urban and rural	Cross-sectional	375	51.83	Cannot retrieve	Quantitative
Dahl H (75)	Unpublished	Population	Zambia	Low	18 July 2018 - 24 January 2019	Urban and rural	Cross-sectional	1048	83	Stratified	Quantitative
Gebrehiwot EM et al., 2015 (32)	Published	Population	Ethiopia	Low	January and February 2011	Urban and rural	Cross-sectional	413	60.4	Random	Quantitative
Ekpo O, 2016 (63)	Published	Population	Nigeria	Lower middle	Cannot retrieve	Urban and rural	Cross-sectional	282	50.4	Random	Quantitative
Azage M et al., 2015 (33)	Published	Population	Ethiopia	Low	Cannot retrieve	Urban and rural	Cross-sectional	1620	35	Stratified cluster	Quantitative

Geda NR et al., 2021 (34)	Published	Population	Ethiopia	Low	Cannot retrieve	Urban and rural	Cross-sectional	1227	43	Stratified cluster	Quantitative
Hillow HS et al. (69)	Unpublished	Population	Somalia	Low	Cannot retrieve	Semi urban	Cross-sectional	200	41	Random	Both qualitative and quantitative
Olson CK et al., 2011 (50)	Published	Population	Kenya	Lower middle	2007	Urban and rural	Cross-sectional	760	62.58	Systematic	Quantitative
Muhumuza J et al., 2017 (72)	Published	Population	Uganda	Low	Cannot retrieve	Rural	Cross-sectional	916	82	Systematic	Both qualitative and quantitative
Ogban GI et al., 2020 (64)	Published	Population	Nigeria	Lower middle	10th November 2018 -18th April, 2019	Semi urban	Cross-sectional	314	14.3	Random	Both qualitative and quantitative
Page A-L et al., 2011 (62)	Published	Population	Niger	Low	21st - 28th of May 2009	Rural	Cross-sectional	1066	70.4	Stratified Cluster	Quantitative
Ogbo PU et al., 2019 (17)	Published	Health facility	Nigeria	Lower middle	July - December 2017	Semi urban	Cross-sectional	1197	15.8	Consecutive	Quantitative
YU J-X et al., 2017 (28)	Published	Population	China	Upper middle	October 2nd - November 3rd 2014	Urban	Cross-sectional	110	48.18	Convenience	Quantitative
Malhotra N et al., 2013 (45)	Published	Population	India	Lower middle	November 2005 - August 2006	Urban and rural	Cross-sectional	3890	60	Stratified Cluster	Quantitative
Sreeramareddy CT et al., 2012 (18)	Published	Health facility	India	Lower middle	November 2005 - August 2006	Urban and rural	Cross-sectional	4438	63.1	Stratified Cluster	Quantitative
Adane M et al., 2017 (77)	Published	Population	Ethiopia	Low	October 2014 (first round), November to December 2014 (second round), January 2015 (third round), April 2015 (fourth round), and July 2015 (fifth round)	Urban	Cross-sectional	452	56.6	Multistage	Quantitative

Tshuma B et al. (19)	Unpublished	Health facility	Botswana	Upper middle	March – April 2018	Rural	Cross-sectional	238	40.3	Systematic random	Quantitative
Das SK et al., 2013 (23)	Published	Population	Bangladesh	Lower middle	May–June 2007	Rural	Case-control	1128	22.1	Random	Quantitative
Woldeamanuel BT, 2020 (78)	Published	Population	Ethiopia	Low	January 18, 2016 - June 27, 2016	Urban and rural	Cross-sectional	9941	13	Stratified two stages	Quantitative
Woldeamanuel BT, 2020 (78)	Published	Population	Ethiopia	Low	Early February – mid-June 2000	Urban and rural	Cross-sectional	8762	44		Quantitative
Benova L et al., 2015 (29)	Published	Population	Egypt	Lower middle	2008	Urban rural semi urban	Cross-sectional	10006	62	Multi-stage stratified	Quantitative
Alene M et al., 2019 (35)	Published	Population	Ethiopia	Low	January 18, 2016 – June 27, 2016	Rural	Cross-sectional	1576	49.7	Stratified Cluster	Quantitative
Basa S, 2015 (46)	Published	Population	India	Lower middle	25th – 31st May, 2007	Urban	Cross-sectional	695	13.96	NS	Quantitative
Bayham M et al., 2017 (59)	Published	Population	Mozambique	Low	April – May 2014	Urban and rural	Cross-sectional	2317	57	Two-stage cluster sampling	Quantitative
Wilson SE et al., 2012 (25)	Published	Population	Burkina Faso	Low	April – September, 2010	Rural	Cross-sectional	10292	77.5	Two-stage procedure	Quantitative
Gebretsadik A et al., 2015 (36)	Published	Population	Ethiopia	Low	2011	Urban and rural	Cross-sectional	2842	35.56	Stratified Cluster	Quantitative
Shah S et al., 2019 (61)	Published	Population	Nepal	Lower middle	Cannot retrieve	Urban	Cross-sectional	117	90.5	Random	Quantitative
Breiman RF et al., 2011 (51)	Published	Population	Kenya	Lower middle	July 2005	Urban	Cross-sectional	2778	52.5	Census	Quantitative
Burton DC et al., 2011 (52)	Published	Population	Kenya	Lower middle	August 2005	Rural	Cross-sectional	1679	36	Cluster	Quantitative
Doracaj D et al, 2015 (20)	Published	Population	Albania	Upper middle	June – July 2012	Rural	Cross-sectional	600	61	Two-stage 30-cluster	Quantitative
Dey (Pal) I et al., 2012 (47)	Published	Population	India	Lower middle	Cannot retrieve	Rural	Cross-sectional	227	90.7	All children were included	Quantitative

Motlagh ME et al., 2012 (49)	Published	Population	Iran	Lower middle	2006	Urban and rural	Cross-sectional	14625	70	Multi-stage stratified and Quota random sampling	Quantitative
Nhampossa T et al., 2013 (60)	Published	Population	Mozambique	Low	May 8 – June 28, 2007	Rural	Cross-sectional	1059	65.2	Stratified random	Quantitative
Nhampossa T et al., 2013 (60)	Published	Population	Mozambique	Low	February 16, 2009 –December 30, 2010	Rural	Cross-sectional	1059	43.8	Stratified random	Quantitative
Omore R et al., 2013 (53)	Published	Population	Kenya	Lower middle	May 22, 2009 – December 31, 2010	Rural and Urban	Cross-sectional	214	57.48	Random	Quantitative
Farag TH et al., 2013 (58)	Published	Population	Mali	Low	March 9 – May 12, 2007	Urban	Cross-sectional	1000	27.3	Random	Quantitative
Saha D et al., 2013 (42)	Published	Population	Gambia	Low	2009 and 2010	Rural	Cross-sectional	1012	81.5	Random	Quantitative
Nasrin D et al., 2013 (24)	Published	Population	Gambia	Low	Baseline in 2007 and January 2009 to March 2011	Rural	Case-control	258	49.6	Random	Quantitative
Nasrin D et al., 2013 (24)	Published	Population	Mali	Lower middle	Baseline in 2007 and January	Urban	Case-control	126	25.1	Random	Quantitative
Nasrin D et al., 2013 (24)	Published	Population	Mozambique	Low	2009 to March 2011	Rural	Case-control	67	84.3	Random	Quantitative
Nasrin D et al., 2013 (24)	Published	Population	Kenya	Lower middle	Baseline in 2007 and January	Rural	Case-control	275	35.1	All included	Quantitative
Nasrin D et al., 2013 (24)	Published	Population	India	Lower middle	2009 to March 2011	Urban	Case-control	92	15.8	Random	Quantitative
Nasrin D et al., 2013 (24)	Published	Population	Bangladesh	Lower middle	Baseline in 2007 and January	Rural	Case-control	95	13.2	Random	Quantitative
Nasrin D et al., 2013 (24)	Published	Population	Pakistan	Lower middle	2009 to March 2011	Peri-urban	Case-control	349	29.8	Random	Quantitative
Biresaw Nega et al. (37)	Unpublished	Population	Ethiopia	Low	Cannot retrieve	Urban and rural	Cross-sectional	624	77.7	Cannot retrieve	Quantitative
Lanyero H et al., 2021 (73)	Published	Population	Uganda	Low	November 1,2018 – December 1,2018	Rural	Cross-sectional	318	38.68	Systematic random	Quantitative

Nyasulu PS et al., 2019 (56)	Published	Population	Malawi	Low	2015 – 2016	Urban and rural	Cross-sectional	3584	59.18	Proportional allocation	Quantitative
Wanjiru AM et al. (54)	Unpublished	Population	Kenya	Lower middle	Cannot retrieve	Urban and rural	Cross-sectional	56	58.9	Multistage	Both qualitative and quantitative
Musuka G, 2021 (76)	Published	Population	Zimbabwe	Lower middle	Last quarter of 2015 – first quarter of 2016	Urban and rural	Cross-sectional	931	41.68	Multistage	Quantitative
Idowu A et al., 2020 (65)	Published	Population	Nigeria	Lower middle	May-June 2019	Rural	Cross-sectional	252	32.9	Multistage	Quantitative
Colombara DV et al., 2016 (30)	Published	Population	Guatemala	Lower middle	April 2013 – August 2013	Urban and rural	Cross-sectional	603	70.3	Multistage	Quantitative
Colombara DV et al., 2016 (30)	Published	Population	Mexico	Lower middle	July 2012 – May 2013	Urban and rural	Cross-sectional	545	67	Multistage	Quantitative
Colombara DV et al., 2016 (30)	Published	Population	Nicaragua	Lower middle	March 2013 – August 2013	Urban and rural	Cross-sectional	191	78.8	Cannot retrieve	Quantitative
Colombara DV et al., 2016 (30)	Published	Population	Panama	Lower middle	April 2013 – August 2013	Rural	Cross-sectional	99	90.8	Cannot retrieve	Quantitative
Colombara DV et al., 2016 (30)	Published	Population	El Salvador	Lower middle	March 2011 – July 2011	Urban and rural	Cross-sectional	497	87.1	Cannot retrieve	Quantitative
Manna B et al., 2013 (48)	Published	Population	India	Lower middle	February 2009 – December 2010	Urban	Case-control	92	47.6	Cannot retrieve	Quantitative
Manna B et al., 2013 (48)	Published	Population	India	Lower middle	April – August 2007	Urban	Case-control	424	13.5	Cannot retrieve	Quantitative
Lungu EA et al., 2020 (57)	Published	Population	Malawi	Low	October 2011 – February, 2013	Urban	Cross-sectional	193	66.32	Cannot retrieve	Quantitative
Kenny A et al., 2015 (55)	Published	Population	Liberia	Low	August–September 2012	Rural	Cross-sectional	559	50.2	Random	Quantitative
Diouf K et al., 2014 (26)	Published	Population	Burundi	Low	June-July 2011	Rural	Cross-sectional	294	70.41	Cluster	Quantitative
Nkouabi JI et al., 2018 (70)	Published	Population	Tanzania	Lower middle	August 2015 – May 2016	Urban and rural	Cross-sectional	279	11.5	Multistage	Quantitative
Bagbi BM et al., 2014 (66)	Published	Population	Nigeria	Lower middle	Cannot retrieve	Urban and rural	Cross-sectional	29	86.21	Cannot retrieve	Quantitative
Kanté AM et al., 2015 (71)	Published	Population	Tanzania	Lower middle	May – June 2011	Rural	Cross-sectional	250	19.6	Cannot retrieve	Quantitative
Kolola T et al., 2016 (38)	Published	Population	Ethiopia	Low	January – February 2011	Urban and rural	Cross-sectional	259	81.08	Cannot retrieve	Quantitative

Bellete M et al., 2021 (79)	Published	Population	Ethiopia	Low	January to February 2020	Urban	Cross-sectional	297	79.46	Multistage	Quantitative
Gelaw YA et al., 2014 (40)	Published	Population	Ethiopia	Low	April to June, 2013 201	Urban and rural	Comparative cross – sectional	347	83	Multistage	Quantitative
Demelash D et al., 2020 (41)	Published	Population	Ethiopia	Low	February 28 to March 28,2019	Urban	Cross-sectional	293	60.07	Random	Quantitative
Demissie B et al., 2014 (39)	Published	Population	Ethiopia	Low	January 2012	Urban and rural	Cross-sectional	349	49.86	Random	Quantitative

Table 3: Characteristics of studies included in the systematic review and meta-analysis of treatment seeking practices of caregivers for children < 5 years with diarrhea in low and middle-income countries, 2010–2022.

Author (reference)	Publication Status	Study Setting	Study Country	World Bank category	Study Period	Residence	Study Design	Sample	Prevalence (treatment seeking)	Sampling Method	Methodology
Ahmed S et al., 2020 (21)	Published	Population	Bangladesh	Lower middle	2019	Urban and rural	Cross-sectional	1598	28.00	Stratified cluster	Quantitative
Diaz T et al., 2013 (68)	Published	Populationca	Sierra Leone	Lower middle	June and July 2010 quantitative and April 2010 and later in July 2010 Qualitative	Rural	Cross-sectional	5951	87.00	Random	Both qualitative and quantitative
Aggarwal AK et al., 2018 (44)	Published	Population	India	Lower middle	October 2013 and April 2014	Urban and rural	Cross-sectional	262	72.8	Random	Quantitative
Fissehaye T et al., 2018 (31)	Published	Population	Ethiopia	Low	March 2015 to June, 2016	Urban	Cross-sectional		72.5	Systematic	Quantitative
Bruce N et al., 2014 (43)	Published	Population	Guatemala	Upper middle	2 surveys from October 2008 to May 2009, and June to December 2009	Rural	Cross-sectional	481	30.6	Cannot retrieve	Quantitative
Gao W et al., 2012 (27)	Published	Population	China	Upper middle	June - August 2005	Semi urban	Cross-sectional	1040	76.54	Random	Quantitative
Hamooya BM et al., 2020 (74)	Published	Population	Zambia	Low	July 2006 - June 2007	Rural	Cross-sectional	1212	32.0	Systematic	Quantitative
Quadri F et al., 2013 (67)	Published	Population	Pakistan	Lower middle	April 2009 - March 2011	Semi urban	Cross-sectional	349	80.3	Random	Quantitative
Sarker AR et al., 2016 (22)	Published	Population	Bangladesh	Lower middle	June 28, 2014 - November 9, 2014	Urban and rural	Cross-sectional	375	51.83	Cannot retrieve	Quantitative
Dahl H (75)	Unpublished	Population	Zambia	Low	18 July 2018 - 24 January 2019	Urban and rural	Cross-sectional	1048	83.0	Stratified	Quantitative
Gebrehiwot EM et al., 2015 (32)	Published	Population	Ethiopia	Low	January and February 2011	Urban and rural	Cross-sectional	413	60.4	Random	Quantitative
Ekpo O, 2016 (63)	Published	Population	Nigeria	Lower middle	Cannot retrieve	Urban and rural	Cross-sectional	282	50.4	Random	Quantitative
Azage M et al., 2015 (33)	Published	Population	Ethiopia	Low	Cannot retrieve	Urban and rural	Cross-sectional	1620	35.0	Stratified cluster	Quantitative

Geda NR et al., 2021 (34)	Published	Population	Ethiopia	Low	Cannot retrieve	Urban and rural	Cross-sectional	1227	43.0	Stratified cluster	Quantitative
Hillow HS et al. (69)	Unpublished	Population	Somalia	Low	Cannot retrieve	Semi urban	Cross-sectional	200	41.0	Random	Both qualitative and quantitative
Olson CK et al., 2011 (50)	Published	Population	Kenya	Lower middle	2007	Urban and rural	Cross-sectional	760	62.58	Systematic	Quantitative
Muhumuza J et al., 2017 (72)	Published	Population	Uganda	Low	Cannot retrieve	Rural	Cross-sectional	916	82.0	Systematic	Both qualitative and quantitative
Ogban GI et al., 2020 (64)	Published	Population	Nigeria	Lower middle	10th November 2018 -18th April, 2019	Semi urban	Cross-sectional	314	14.3	Random	Both qualitative and quantitative
Page A-L et al., 2011 (62)	Published	Population	Niger	Low	21st - 28th of May 2009	Rural	Cross-sectional	1066	70.4	Stratified Cluster	Quantitative
Ogbo PU et al., 2019 (17)	Published	Health facility	Nigeria	Lower middle	July - December 2017	Semi urban	Cross-sectional	1197	15.8	Consecutive	Quantitative
YU J-X et al., 2017 (28)	Published	Population	China	Upper middle	October 2nd - November 3rd 2014	Urban	Cross-sectional	110	48.18	Convenience	Quantitative
Malhotra N et al., 2013 (45)	Published	Population	India	Lower middle	November 2005 - August 2006	Urban and rural	Cross-sectional	3890	60.0	Stratified Cluster	Quantitative
Sreeramareddy CT et al., 2012 (18)	Published	Health facility	India	Lower middle	November 2005 - August 2006	Urban and rural	Cross-sectional	4438	63.1	Stratified Cluster	Quantitative
Adane M et al., 2017 (77)	Published	Population	Ethiopia	Low	October 2014 (first round), November to December 2014 (second round), January 2015 (third round), April 2015 (fourth round), and July 2015 (fifth round)	Urban	Cross-sectional	452	56.6	Multistage	Quantitative

Tshuma B et al. (19)	Unpublished	Health facility	Botswana	Upper middle	March – April 2018	Rural	Cross-sectional	238	40.3	Systematic random	Quantitative
Das SK et al., 2013 (23)	Published	Population	Bangladesh	Lower middle	May–June 2007	Rural	Case-control	1128	22.1	Random	Quantitative
Woldeamanuel BT, 2020 (78)	Published	Population	Ethiopia	Low	January 18, 2016 - June 27, 2016	Urban and rural	Cross-sectional	9941	13.0	Stratified two stages	Quantitative
Woldeamanuel BT, 2020 (78)	Published	Population	Ethiopia	Low	Early February – mid-June 2000	Urban and rural	Cross-sectional	8762	44.0		Quantitative
Benova L et al., 2015 (29)	Published	Population	Egypt	Lower middle	2008	Urban rural semi urban	Cross-sectional	10006	62.0	Multi-stage stratified	Quantitative
Alene M et al., 2019 (35)	Published	Population	Ethiopia	Low	January 18, 2016 – June 27, 2016	Rural	Cross-sectional	1576	49.7	Stratified Cluster	Quantitative
Basa S, 2015 (46)	Published	Population	India	Lower middle	25th – 31st May, 2007	Urban	Cross-sectional	695	13.96	NS	Quantitative
Bayham M et al., 2017 (59)	Published	Population	Mozambiq ue	Low	April – May 2014	Urban and rural	Cross-sectional	2317	57.0	Two-stage cluster sampling	Quantitative
Wilson SE et al., 2012 (25)	Published	Population	Burkina Faso	Low	April – September, 2010	Rural	Cross-sectional	10292	77.5	Two-stage procedure	Quantitative
Gebretsadik A et al., 2015 (36)	Published	Population	Ethiopia	Low	2011	Urban and rural	Cross-sectional	2842	35.56	Stratified Cluster	Quantitative
Shah S et al., 2019 (61)	Published	Population	Nepal	Lower middle	Cannot retrieve	Urban	Cross-sectional	117	90.5	Random	Quantitative
Breiman RF et al., 2011 (51)	Published	Population	Kenya	Lower middle	July 2005	Urban	Cross-sectional	2778	52.5	Census	Quantitative
Burton DC et al., 2011 (52)	Published	Population	Kenya	Lower middle	August 2005	Rural	Cross-sectional	1679	36.0	Cluster	Quantitative
Doracaj D et al, 2015 (20)	Published	Population	Albania	Upper middle	June – July 2012	Rural	Cross-sectional	600	61.0	Two-stage 30-cluster	Quantitative
Dey (Pal) I et al., 2012 (47)	Published	Population	India	Lower middle	Cannot retrieve	Rural	Cross-sectional	227	90.7	All children were included	Quantitative

Motlagh ME et al., 2012 (49)	Published	Population	Iran	Lower middle	2006	Urban and rural	Cross-sectional	14625	70.0	Multi-stage stratified and Quota random sampling	Quantitative
Nhampossa T et al., 2013 (60)	Published	Population	Mozambique	Low	May 8 – June 28, 2007	Rural	Cross-sectional	1059	65.2	Stratified random	Quantitative
Nhampossa T et al., 2013 (60)	Published	Population	Mozambique	Low	February 16, 2009 –December 30, 2010	Rural	Cross-sectional	1059	43.8	Stratified random	Quantitative
Omore R et al., 2013 (53)	Published	Population	Kenya	Lower middle	May 22, 2009 – December 31, 2010	Rural and Urban	Cross-sectional	214	57.48	Random	Quantitative
Farag TH et al., 2013 (58)	Published	Population	Mali	Low	March 9 – May 12, 2007	Urban	Cross-sectional	1000	27.3	Random	Quantitative
Saha D et al., 2013 (42)	Published	Population	Gambia	Low	2009 and 2010	Rural	Cross-sectional	1012	81.5	Random	Quantitative
Nasrin D et al., 2013 (24)	Published	Population	Gambia	Low	Baseline in 2007 and January 2009 to March 2011	Rural	Case-control	258	49.6	Random	Quantitative
Nasrin D et al., 2013 (24)	Published	Population	Mali	Lower middle	Baseline in 2007 and January	Urban	Case-control	126	25.1	Random	Quantitative
Nasrin D et al., 2013 (24)	Published	Population	Mozambique	Low	2009 to March 2011	Rural	Case-control	67	84.3	Random	Quantitative
Nasrin D et al., 2013 (24)	Published	Population	Kenya	Lower middle	Baseline in 2007 and January	Rural	Case-control	275	35.1	All included	Quantitative
Nasrin D et al., 2013 (24)	Published	Population	India	Lower middle	2009 to March 2011	Urban	Case-control	92	15.8	Random	Quantitative
Nasrin D et al., 2013 (24)	Published	Population	Bangladesh	Lower middle	Baseline in 2007 and January	Rural	Case-control	95	13.2	Random	Quantitative
Nasrin D et al., 2013 (24)	Published	Population	Pakistan	Lower middle	2009 to March 2011	Peri-urban	Case-control	349	29.8	Random	Quantitative
Biresaw Nega et al. (37)	Unpublished	Population	Ethiopia	Low	Cannot retrieve	Urban and rural	Cross-sectional	624	77.7	Cannot retrieve	Quantitative
Lanyero H et al., 2021 (73)	Published	Population	Uganda	Low	November 1,2018 – December 1,2018	Rural	Cross-sectional	318	38.68	Systematic random	Quantitative

Nyasulu PS et al., 2019 (56)	Published	Population	Malawi	Low	2015 – 2016	Urban and rural	Cross-sectional	3584	59.18	Proportional allocation	Quantitative
Wanjiru AM et al. (54)	Unpublished	Population	Kenya	Lower middle	Cannot retrieve	Urban and rural	Cross-sectional	56	58.9	Multistage	Both qualitative and quantitative
Musuka G, 2021 (76)	Published	Population	Zimbabwe	Lower middle	Last quarter of 2015 – first quarter of 2016	Urban and rural	Cross-sectional	931	41.68	Multistage	Quantitative
Idowu A et al., 2020 (65)	Published	Population	Nigeria	Lower middle	May-June 2019	Rural	Cross-sectional	252	32.9	Multistage	Quantitative
Colombara DV et al., 2016 (30)	Published	Population	Guatemala	Lower middle	April 2013 – August 2013	Urban and rural	Cross-sectional	603	70.3	Multistage	Quantitative
Colombara DV et al., 2016 (30)	Published	Population	Mexico	Lower middle	July 2012 – May 2013	Urban and rural	Cross-sectional	545	67.0	Multistage	Quantitative
Colombara DV et al., 2016 (30)	Published	Population	Nicaragua	Lower middle	March 2013 – August 2013	Urban and rural	Cross-sectional	191	78.8	Cannot retrieve	Quantitative
Colombara DV et al., 2016 (30)	Published	Population	Panama	Lower middle	April 2013 – August 2013	Rural	Cross-sectional	99	90.8	Cannot retrieve	Quantitative
Colombara DV et al., 2016 (30)	Published	Population	El Salvador	Lower middle	March 2011 – July 2011	Urban and rural	Cross-sectional	497	87.1	Cannot retrieve	Quantitative
Manna B et al., 2013 (48)	Published	Population	India	Lower middle	February 2009 – December 2010	Urban	Case-control	92	47.6	Cannot retrieve	Quantitative
Manna B et al., 2013 (48)	Published	Population	India	Lower middle	April – August 2007	Urban	Case-control	424	13.5	Cannot retrieve	Quantitative
Lungu EA et al., 2020 (57)	Published	Population	Malawi	Low	October 2011 – February, 2013	Urban	Cross-sectional	193	66.32	Cannot retrieve	Quantitative
Kenny A et al., 2015 (55)	Published	Population	Liberia	Low	August–September 2012	Rural	Cross-sectional	559	50.2	Random	Quantitative
Diouf K et al., 2014 (26)	Published	Population	Burundi	Low	June-July 2011	Rural	Cross-sectional	294	70.41	Cluster	Quantitative
Nkouabi JI et al., 2018 (70)	Published	Population	Tanzania	Lower middle	August 2015 – May 2016	Urban and rural	Cross-sectional	279	11.5	Multistage	Quantitative
Bagbi BM et al., 2014 (66)	Published	Population	Nigeria	Lower middle	Cannot retrieve	Urban and rural	Cross-sectional	29	86.21	Cannot retrieve	Quantitative
Kanté AM et al., 2015 (71)	Published	Population	Tanzania	Lower middle	May – June 2011	Rural	Cross-sectional	250	19.6	Cannot retrieve	Quantitative
Kolola T et al., 2016 (38)	Published	Population	Ethiopia	Low	January – February 2011	Urban and rural	Cross-sectional	259	81.08	Cannot retrieve	Quantitative

Bellete M et al., 2021 (79)	Published	Population	Ethiopia	Low	January to February 2020	Urban	Cross-sectional	297	79.46	Multistage	Quantitative
Gelaw YA et al., 2014 (40)	Published	Population	Ethiopia	Low	April to June, 2013 201	Urban and rural	Comparative cross – sectional	347	83.0	Multistage	Quantitative
Demelash D et al., 2020 (41)	Published	Population	Ethiopia	Low	February 28 to March 28,2019	Urban	Cross-sectional	293	60.07	Random	Quantitative
Demissie B et al., 2014 (39)	Published	Population	Ethiopia	Low	January 2012	Urban and rural	Cross-sectional	349	49.86	Random	Quantitative
